# Health Care Utilization Following Interventions to Improve Social Well-Being: A Systematic Review and Meta-analysis

**DOI:** 10.1001/jamanetworkopen.2023.21019

**Published:** 2023-06-29

**Authors:** Neta HaGani, Daniel L. Surkalim, Philip J. Clare, Dafna Merom, Ben J. Smith, Ding Ding

**Affiliations:** 1 Prevention Research Collaboration, Sydney School of Public Health, The University of Sydney, Sydney, Australia; 2 National Drug and Alcohol Research Centre, UNSW Sydney, Sydney, Australia; 3 School of Health Science, Western Sydney University, Sydney, Australia; 4 Charles Perkins Centre, the University of Sydney, Sydney, Australia

## Abstract

**Importance:**

It has been suggested that interventions that aim to improve social well-being may contribute to decreased health care use; however, such evidence has not been fully systematically synthesized.

**Objective:**

To systematically review and meta-analyze available evidence on the associations between psychosocial interventions and health care utilization.

**Data Sources:**

Medline, Embase, PsycInfo, Cumulated Index to Nursing and Allied Health Literature, Cochrane, Scopus, Google Scholar, and reference lists of systematic reviews were searched from inception until November 31, 2022.

**Study Selection:**

Included studies were randomized clinical trials reporting on both health care utilization and social well-being outcomes.

**Data Extraction and Synthesis:**

The reporting of the systematic review adhered to the Preferred Reporting Items for Systematic Reviews and Meta-analyses (PRISMA) reporting guideline. Full-text and quality assessments were conducted by 2 reviewers independently. Multilevel random-effects meta-analyses were used to synthesize the data. Subgroup analyses were conducted to examine the characteristics associated with decreased health care use.

**Main Outcomes and Measures:**

The outcome of interest was health care utilization, including primary, emergency, inpatient, and outpatient care services. Social well-being was measured as social support, social participation, social relationships, community support, social integration, or loneliness.

**Results:**

A total of 41 studies were retrieved from 18 969 citations; 37 studies were eligible for meta-analysis. Data were analyzed for 7842 participants, including 2745 older adults, 1579 young women considered to be at risk of social and mental health disadvantages, 1118 people with chronic illnesses, 1597 people with mental illnesses, and 803 caregivers. The odds ratio (OR) random-effects model showed an overall reduction in health care use (OR, 0.75; 95% CI, 0.59 to 0.97), but the standardized mean difference (SMD) random effect model showed no association. An improvement in health care utilization was observed in association with social support interventions (SMD, 0.25; 95% CI, 0.04 to 0.45) but not in loneliness interventions. Subgroup analysis indicated a reduced length of inpatient visits (SMD, −0.35; 95% CI, −0.61 to −0.09) and number of emergency care visits (OR, 0.64; 95% CI, 0.43 to 0.96) following the intervention. However, an increase in outpatient care associated with psychosocial interventions was observed (SMD, 0.34; 95% CI, 0.05 to 0.62). The largest reductions in health care use were associated with interventions among caregivers (OR, 0.23; 95% CI, 0.07 to 0.71) and individuals with mental illnesses (OR, 0.31; 95% CI, 0.13 to 0.74).

**Conclusions:**

These findings suggest that psychosocial interventions were associated with most measures of health care utilization. As the association differed by participant and intervention delivery characteristics, these characteristics should be considered in the design of future interventions.

## Key Points

**Question:**

Is there an association between psychosocial interventions and health care service use, and does the association differ by sociodemographic or intervention characteristic?

**Findings:**

This systematic review and meta-analysis including 41 studies and 7842 participants found that psychosocial interventions were associated with decreased health care use in most health services and increased use of outpatient care. The greatest health care decrease was among caregivers and individuals with mental illnesses and in interventions delivered 1-on-1 by health professionals.

**Meaning:**

These findings suggest that psychosocial interventions were associated with reduced health care use.

## Teaser

This systematic review and meta-analysis explores associations between psychosocial interventions and health care utilization.

## Introduction

*Social well-being* is an umbrella term that refers to the actual or perceived availability of social resources, such as social networks.^1^ A lack of social well-being can manifest in problems, such as loneliness and social isolation, that are important public health concerns^2,3,4^ and can lead to chronic diseases and premature mortality.^5,6,7^

Another public health challenge faced by many countries is escalating health care costs associated with population aging and increasing prevalence of chronic disease. As this trend is expected to continue,^8^ decision-makers need to find solutions to reduce health care spending by designing and implementing efficient and equitable health services and minimizing unnecessary health care use. Promising evidence from observational studies has suggested that better social well-being is associated with lower health care utilization,^9,10,11,12^ indicating that psychosocial interventions to address social well-being may reduce health care demand.^13,14^

To date, psychosocial interventions have been conducted among a variety of populations^15,16^ using myriad approaches, such as individual or group therapy, group-based activities, peer support, and outreach and befriending strategies.^17,18^ Besides positive associations with loneliness, social interaction, and perceptions of support,^19,20^ some psychosocial interventions have been found to be associated with decreases in unnecessary health service use and costs in some populations, such as older adults, survivors of breast cancer, and people with mental illnesses.^17,18,21^ For example, peer support interventions and multicomponent psychosocial interventions were associated with decreased use of general practitioners and emergency care among verity of populations.^18,22^ However, for outpatient care, such as visits to specialists, most studies showed no significant changes.^22,23,24^

Despite the associations found between psychosocial interventions and health care utilization, evidence on this topic has not been systematically synthesized or quantitatively summarized, to our knowledge.^13^ Furthermore, we do not yet know what intervention characteristics are associated with lower health care use. To address these gaps, we aimed to synthesize the associations among psychosocial interventions, health care utilization, and social well-being and identify characteristics associated with the variability in effect sizes of these outcomes.

## Methods

The reporting of the systematic review and meta-analysis adhered to the Preferred Reporting Items for Systematic Reviews and Meta-analyses (PRISMA) reporting guideline. The protocol is registered in the PROSPERO database under record No. CRD42021273388.

### Eligibility Criteria

Psychosocial interventions that used a randomized clinical trial design to improve social well-being were included. To be eligible, studies needed to report on at least 1 health care utilization–related outcome and at least 1 social well-being–related outcome. *Health care utilization* refers to using primary care (visits to the general practitioner or nurse), emergency care (visits to the emergency department), inpatient care (eg, number of hospitalizations and readmissions, length of stay), and outpatient care (visits to a specialist, such as a cardiologist, obstetrician, or psychiatrist) services. Social well-being covers multiple domains, including social support, social participation, social relationships, community support, and loneliness. The population, intervention, control, and outcomes data are presented in eTable 1 in Supplement 1.

### Study Selection and Data Extraction

A comprehensive search was conducted from inception until May 31, 2021, and an update search until November 31, 2022, in the following databases: Medline, Embase, PsycInfo, Cumulated Index to Nursing and Allied Health Literature, Cochrane, and Scopus. Search terms on the concepts of health care utilization, social well-being, and psychosocial interventions were combined with AND (specific search terms and search results are presented in eTables 2-8 in Supplement 1). Google Scholar, reference lists of the included studies, and relevant systematic reviews^10,13,14,25^ were manually searched for additional potential studies. All records were imported to Covidence reference management software (Veritas Health Innovation). After duplicates were removed, titles and abstracts were assessed according to the eligibility criteria. The full-text review was conducted by 2 reviewers (N.H. and D.S.) independently. Disagreements were resolved by a third reviewer (D.D., D.M., or B.J.S.). Data were extracted by 1 reviewer (N.H.) and a sample of the extracted data (approximately 30%) was reviewed by a second reviewer (D.D.). Where means and SDs were missing, they were estimated from other measures of effect^26^ or imputed from other studies,^22,26,27^ as suggested by Cochrane.^28^

For the main outcome of health care utilization, 2 types of data were extracted for the intervention and control groups: raw means and SDs to calculate standardized mean differences (SMDs) and numbers and percentages of health care users to calculate odds ratios (ORs). Some studies included multiple follow-ups after their intervention. Data were extracted and analyzed from each study for the first follow-up immediately after the intervention and for the last follow-up after the intervention, regardless of specific durations of the follow-ups.

### Risk of Bias

All studies were assessed for risk of bias (ROB) using the *Cochrane Handbook for Systematic Reviews of Interventions, Version 5.1.0*.^29^ Assessment was done using Covidence and RevMan version 5.4 (Cochrane) tools. Studies were assessed by 2 independent reviewers (N.H. and D.L.S.). Conflicts were resolved by consultations with a third reviewer (D.M., B.J.S., or D.D.). Each study was assessed on 7 different domains: random sequence generation, allocation concealment, blinding of participants and personnel, blinding of outcome assessment, incomplete outcome data, selective reporting, and other biases. Each domain received a judgement of low, high, or unclear ROB.

### Publication Bias

Publication bias was examined using funnel plots and an extension of Egger regression test was used to quantify the funnel plot asymmetry.^30^ This included using a measure of effect size precision as a predictor in a meta-regression.^31^ *P* < .05 indicated a substantial asymmetry in the funnel plot that could have been caused by publication bias.

### Assessing the Certainty of the Evidence

Certainty of evidence was assessed for each of the overall outcomes using the Grading of Recommendations Assessment, Development and Evaluation (GRADE) approach.^32,33^ The assessment was performed using the GRADEpro GDT software (McMaster University and Evidence Prime). GRADE domains were assessed according to the level of uncertainty (ie, not serious, serious, or very serious). The overall certainty was categorized as very low, low, moderate, or high. Certainty was downgraded 1 level per limitation, starting from high certainty.^33^

### Statistical Analysis

Results were combined and analyzed using random-effects models. Separate meta-analytic estimates were calculated for studies that reported on SMDs and ORs. To account for multiple dependent effect sizes,^34^ we performed a 3-level meta-analysis. The effect sizes of the overall and different types of health care utilization were calculated for the immediate and sustained outcomes after interventions. Subgroup analyses were used to identify differences according to participant characteristics, such as age, gender, and population groups, and intervention characteristics, including type (individual vs group), duration (number of months), and delivery personnel (health professionals, peers, and activity coordinators). All analyses were performed using the meta and metafor libraries of R software version 4.1.3 (R Project for Statistical Computing). Bayes factors (BFs) were calculated to examine the strength of evidence using an online calculator.^35^ BFs with a value between 3 and 10 indicate moderate relative evidence for the alternative hypothesis; BFs between 0.3 and 3, inconclusive evidence; and BFs less than 0.3, moderate evidence for the null hypothesis.^36^ Expected effect sizes were estimated based on previous meta-analyses on related topics.^14,37^ For the SMD random-effects model, the expected SMD was set to −0.3. Similarly, the expected OR was set to 0.70 (30% reduction). For the alternative hypothesis of the social support measure and outpatient care use, we used an SMD of 0.3 and an OR of 1.5, since they were associated positively with intervention effect size.

*P* values were 2-sided, and statistical significance was set at *P* < .05. The last analysis update was conducted between January 12 and 19, 2023.

## Results

### Systematic Review

**Figure 1.**
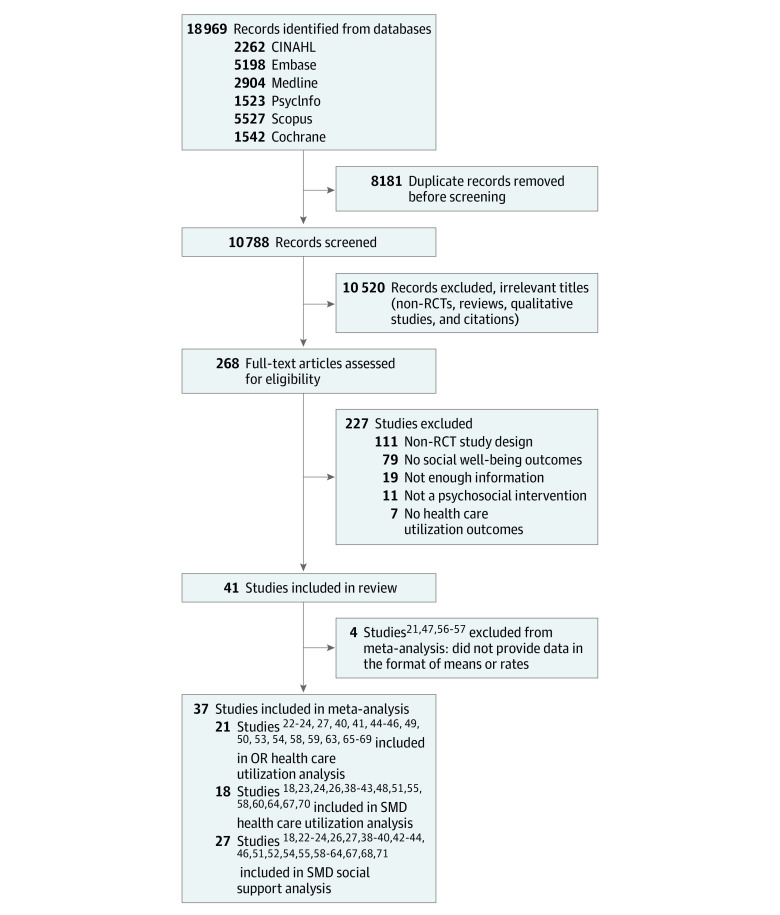
Flowchart of Identification of Studies Via Databases and Registers CINAHL indicates Cumulated Index to Nursing and Allied Health Literature; OR, odds ratio; RCT, randomized clinical trial; SMD, standardized mean difference.

Of 18 968 records identified, 268 full texts articles were assessed for eligibility, 41 studies met the inclusion criteria for the systematic review.^18,21,22,23,24,26,27,38,39,40,41,42,43,44,45,46,47,48,49,50,51,52,53,54,55,56,57,58,59,60,61,62,63,64,65,66,67,68,69,70,71^ Of these, 21 studies^22,23,24,27,40,41,44,45,46,49,50,53,54,58,59,63,65,66,67,68,69^ were included in the OR health care utilization meta-analysis, 18 studies^18,23,24,26,38,39,40,41,42,43,48,51,55,58,60,64,67,70^ were included in the SMD health care utilization meta-analysis, 27 studies^18,22,23,24,26,27,38,39,40,42,43,44,46,51,52,54,55,58,59,60,61,62,63,64,67,68,71^ were included in the social support SMD meta-analysis, and 3 studies^26,40,70^ were included in the loneliness SMD meta-analysis (Figure 1). Data were analyzed for 7842 participants, including 2745 older adults, 1579 young women considered at risk of social and mental health disadvantages (1 study among women in a shelter for family violence survivors, 1 study among women at risk for postpartum depression and 1 study among women from disadvantage living areas), 1118 people with chronic illnesses, 1597 people with mental illnesses, and 803 caregivers.

eTable 9 in Supplement 1 presents the characteristics of the included studies. The included studies were published between 1995 and 2022. Most were conducted in the UK (10 studies^21,23,38,39,41,51,61,67,69,71^) or other European countries (8 studies^18,26,42,48,49,65,66,70^); 6 studies^43,44,54,55,59,60^ were undertaken in China, 7 studies^22,24,27,46,47,52,64^ were undertaken in Canada, 6 studies^38,40,45,50,58,68^ were undertaken in the US, and 1 study each was conducted in Singapore,^62^ Columbia,^63^ Australia,^53^ and Zambia.^57^ Fourteen studies^24,38,39,40,41,42,43,44,45,46,47,48,49,72^ had sample sizes of fewer than 100 participants, 18 studies^18,22,27,50,51,52,53,54,55,56,57,58,59,60,61,62,63,64^ had samples of 100 to 200 participants, 7 studies^23,26,65,66,67,68,69^ included 200 to 1000 participants, and 2 studies^70,71^ had sample sizes of more than 1000 participants. Studied populations included 7 studies^43,47,49,50,53,54,55^ of caregivers, 9 studies of people with physical illnesses (6 on heart disease,^22,41,44,48,51,62^ 1 on HIV,^66^ 1 on stroke,^57^ and 1 on multimorbidity^42^), 11 studies^21,27,45,56,58,59,60,61,63,68,69^ of people with mental illnesses, 7 studies^18,26,39,46,52,65,70^ of older adults, 6 studies^23,24,40,64,67,71^ of postnatal young women, and 1 study^38^ of women at a domestic violence shelter. Interventions were delivered by health professionals (28 studies^18,23,26,38,40,42,43,44,46,47,48,49,50,52,53,54,56,57,58,59,60,62,63,64,65,66,70,71^), peer volunteers (8 studies^21,22,24,27,45,55,68,69^ with individuals with lived experience of health challenges or services), or a coordinator on behalf of the intervention team (5 studies^39,41,51,61,67^). Sixteen interventions^18,26,27,38,39,41,42,49,51,55,60,63,64,65,68,71^ were group-based, 18 interventions^18,21,22,23,24,40,43,44,46,47,52,53,54,56,61,62,66,67,69^ were delivered to individuals 1-on-1, and 7 interventions^45,48,50,57,58,59,70^ included both individual and group components. Most of the control groups received standard care (31 studies^18,21,22,23,24,27,40,41,43,44,45,46,47,48,49,52,53,54,55,56,58,59,60,61,62,63,65,66,67,69,70^). Two studies^26,42^ used a waitlist control, whereas 8 studies^38,39,50,51,57,64,68,71^ had a minimal intervention as a control condition. Seventeen interventions^18,40,41,43,44,46,47,49,51,52,55,60,65,66,67,68,70^ were 6 to 12 months long, and the remainder^18,21,22,23,24,26,27,38,39,42,45,48,50,53,54,56,57,58,59,61,62,63,64,69,71^ were 4 months or shorter.

### Risk of Bias

Sixteen studies^18,21,40,42,46,50,52,54,55,56,60,62,64,66,67,69^ were classified as low risk of bias; 6 studies,^23,26,44,47,49,57^ medium risk; and 19 studies^22,24,27,38,39,41,43,45,48,51,53,58,59,61,63,65,68,70,71^ high risk. Most interventions were classified as low risk for randomization (31 interventions^18,21,22,23,26,27,38,39,40,41,44,46,48,49,50,52,53,54,55,56,57,60,61,62,64,65,67,69,71^ [76%]), allocation concealment (27 interventions^21,22,23,24,26,38,40,41,42,43,44,46,47,49,50,52,54,55,56,57,60,62,64,65,66,67,69^ [66%]) and attrition bias (30 interventions^18,21,22,24,27,40,41,42,43,44,46,47,48,49,50,52,53,54,55,56,58,60,62,63,64,65,67,68,69,70^ [73%]). There were 23 interventions^18,21,23,24,26,39,40,42,46,48,53,54,55,56,57,60,62,63,64,66,67,69,70^ (56%) considered low risk for reporting bias. Only 2 studies^18,21,23,24,26,39,40,42,46,48,53,54,55,56,57,60,62,63,64,66,67,69,70^ (5%) had low risk in the blinding of participants and personnel domain, and 19 studies^18,21,23,24,27,44,46,50,52,54,55,58,60,62,63,64,66,68,69^ (46%) had low risk in the blinding of outcome data domain (eFigure 1 in Supplement 1).

### GRADE Certainty of Evidence Results

The evidence for the immediate postintervention health care utilization and social support outcomes using ORs was evaluated with a moderate level of certainty. The evidence for the sustained health care utilization and loneliness outcomes was assessed with low levels of certainty. Evidence for both immediate and sustained health care utilization outcomes using SMDs was assessed with a very low level of certainty (eTable 10 in Supplement 1).

### Meta-Analyses of Immediate Postintervention Health Care Utilization Outcomes 

**Figure 2.**
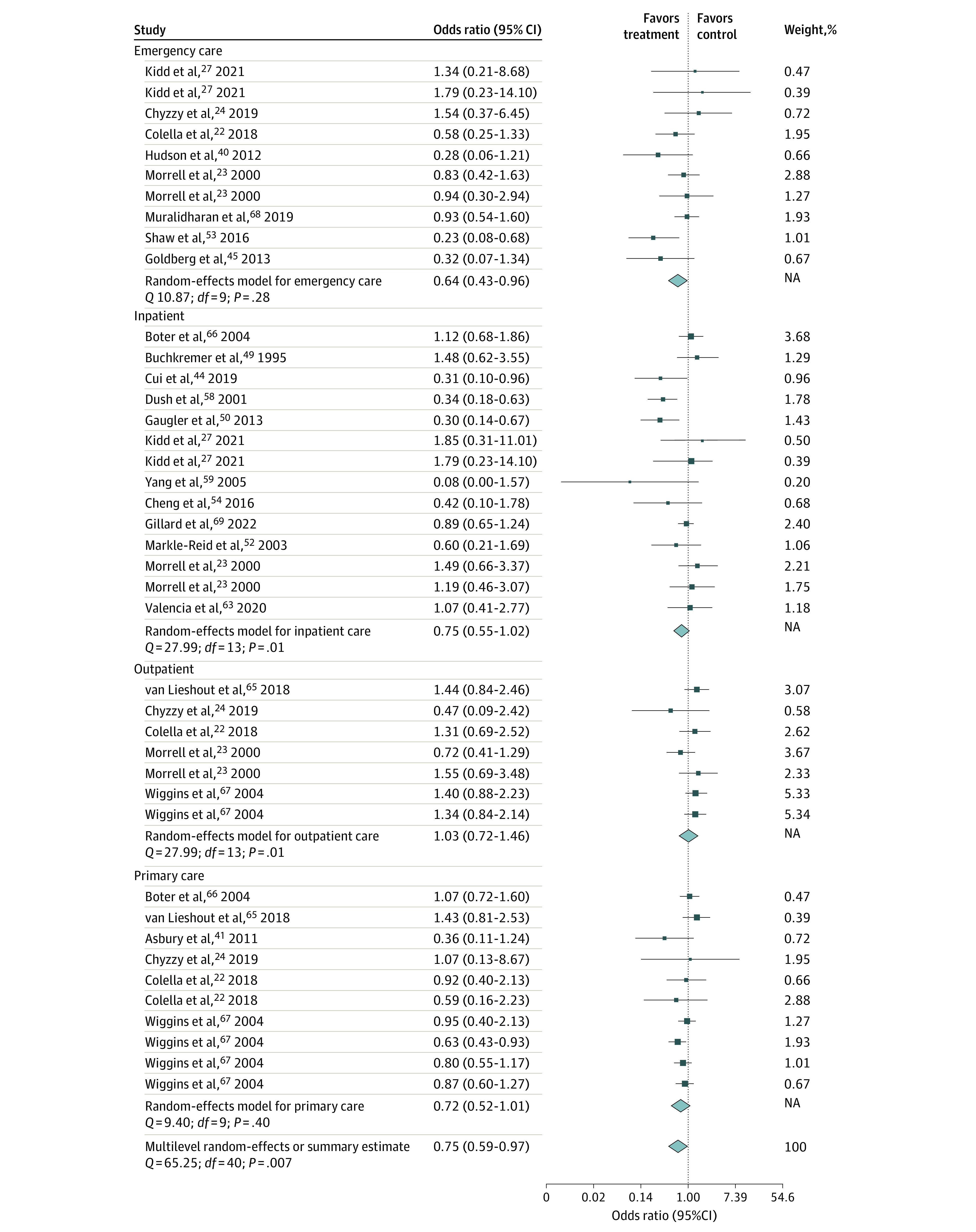
Pooled 95% CI Odds Ratio of the Association of Psychosocial Interventions With Health Care Utilization Since multilevel meta-analyses included several effect estimates from each study, studies may be listed more than once for each analysis. Dots indicate estimates; whiskers, 95% CIs; diamond, summary estimate.

**Figure 3.**
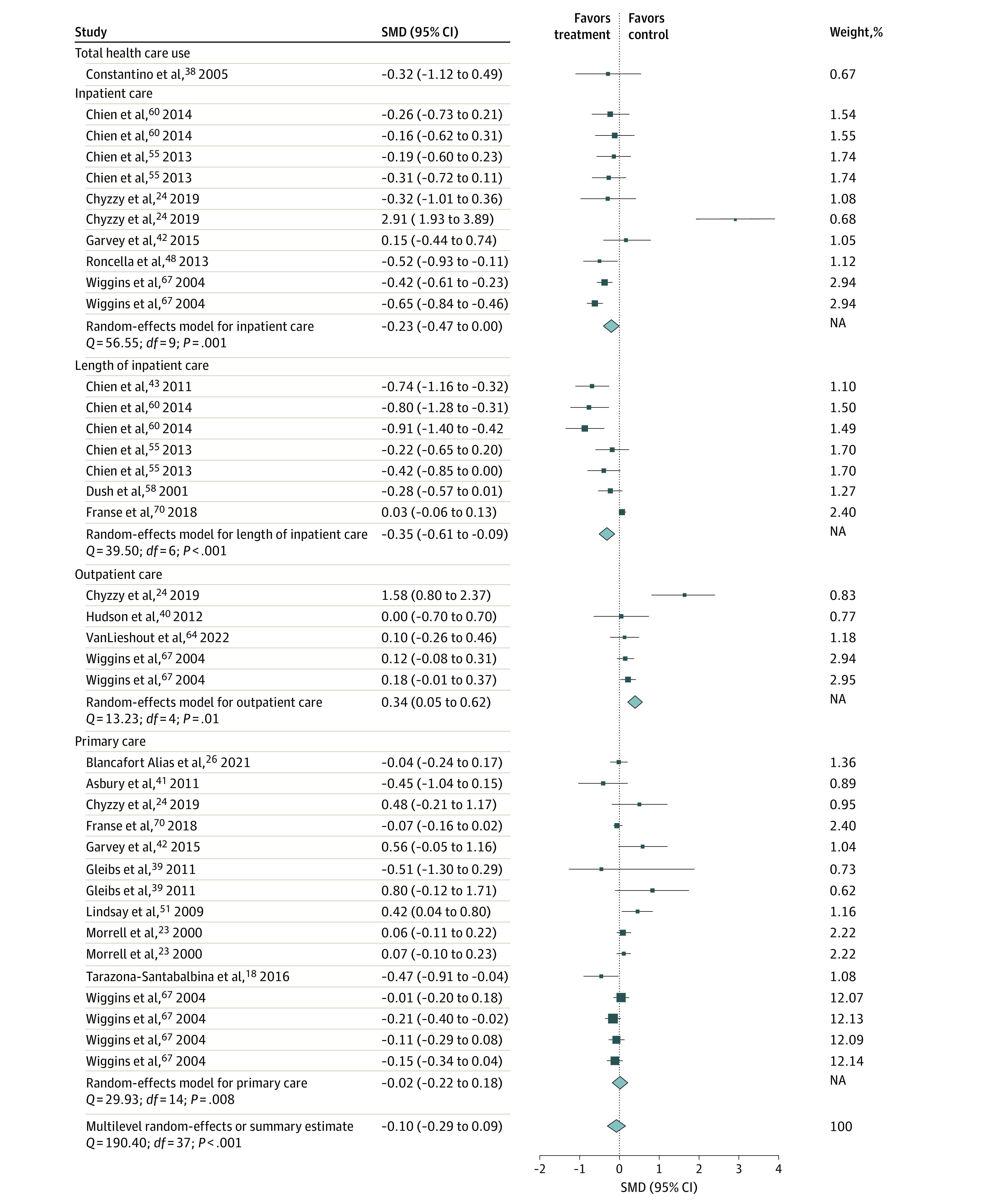
Pooled 95% CI Standardized Mean Difference of the Association of Psychosocial Interventions With Health Care Utilization Since multilevel meta-analyses included several effect estimates from each study, studies may be listed more than once for each analysis. Dots indicate estimates; whiskers, 95% CIs; diamond, summary estimate; SMD, standardized mean difference.

According to the random-effects model of studies reporting ORs, the intervention group had significant lower odds of using health care compared with the control group (OR, 0.75; 95% CI, 0.59 to 0.97). However, the SMD random-effects model was not significant. When looking at different services, the OR model showed sizeable and significant reductions in the odds of emergency care use (OR, 0.64; 95% CI, 0.43-0.96) among the intervention groups. The SMD model showed significant increase in outpatient care (SMD, 0.34; 95% CI, 0.05-0.62), a decrease in length of inpatient care (SMD, −0.35; 95% CI, −0.61 to −0.09) by the intervention group. Heterogeneity was significant for both the OR and SMD random-effects models, suggesting that the amount of between-study variability was greater than would be expected by chance (Figure 2 and Figure 3).

### Bayes Factors

The strength of evidence was moderate for the overall OR random-effects model (BF = 5.63) and was inconclusive for the overall SMD random-effects model (BF = 0.84). Strong relative evidence for the alternative hypothesis was found in the SMD model for inpatient length (BF > 10) and moderate evidence was found for inpatient and outpatient services utilization (BF, 3-10). In the OR models, we found moderate evidence for the alternative hypothesis for primary care, inpatient, and emergency care. We also found moderate evidence for the alternative hypothesis for social support in the SMD model (BF = 7.21) (eTable 11 in Supplement 1).

### Subgroup Analysis

In both the OR and SMD models, we observed significant decreases in health care use after interventions in studies conducted in China compared with other countries.^43,44,45,46,47,48,49,50,51,52,53,54,55,59,60^ In the OR model only, we observed significant decreases in health care use after interventions in studies conducted in the US and Australia.^40,45,50,53,58,68^ In the OR model, we also observed a decrease in health care use in studies that included participants aged 30 to 60 years.^27,44,45,50,53,54,59,68,69^ Reductions in health care use were observed in studies among caregivers^49,50,53,54^ and individuals with mental illnesses^27,45,58,59,63,68,69^ compared with other population groups, but only in the OR model. Single-component interventions that were based on the individual level, shorter (1-4 months), and delivered by health professionals were associated with significant decreases in health care use as well. However, in the SMD model, longer interventions were significantly associated with lower health care use after the interventions (eTable 12 in Supplement 1).

### Meta-Analyses of Sustained Postintervention Health Care Utilization Outcomes

Measures of effect were calculated for the last follow-up measure in a subset of 5 studies^22,23,54,59,67^ that included ORs and 5 studies23,61,66,70,73 that included 13 SMDs that included more than 1 follow-up (eFigure 3 and eFigure 4 in Supplement 1). The OR random-effects model showed a significant decrease only in inpatient care (OR, 0.52; 95% CI, 0.28 to 0.98) and the SMD model showed a lower length of inpatient care (SMD, −1.21; 95% CI, −1.67 to −0.75).

### Meta-Analyses of Social Support and Loneliness Outcomes

**Figure 4.**
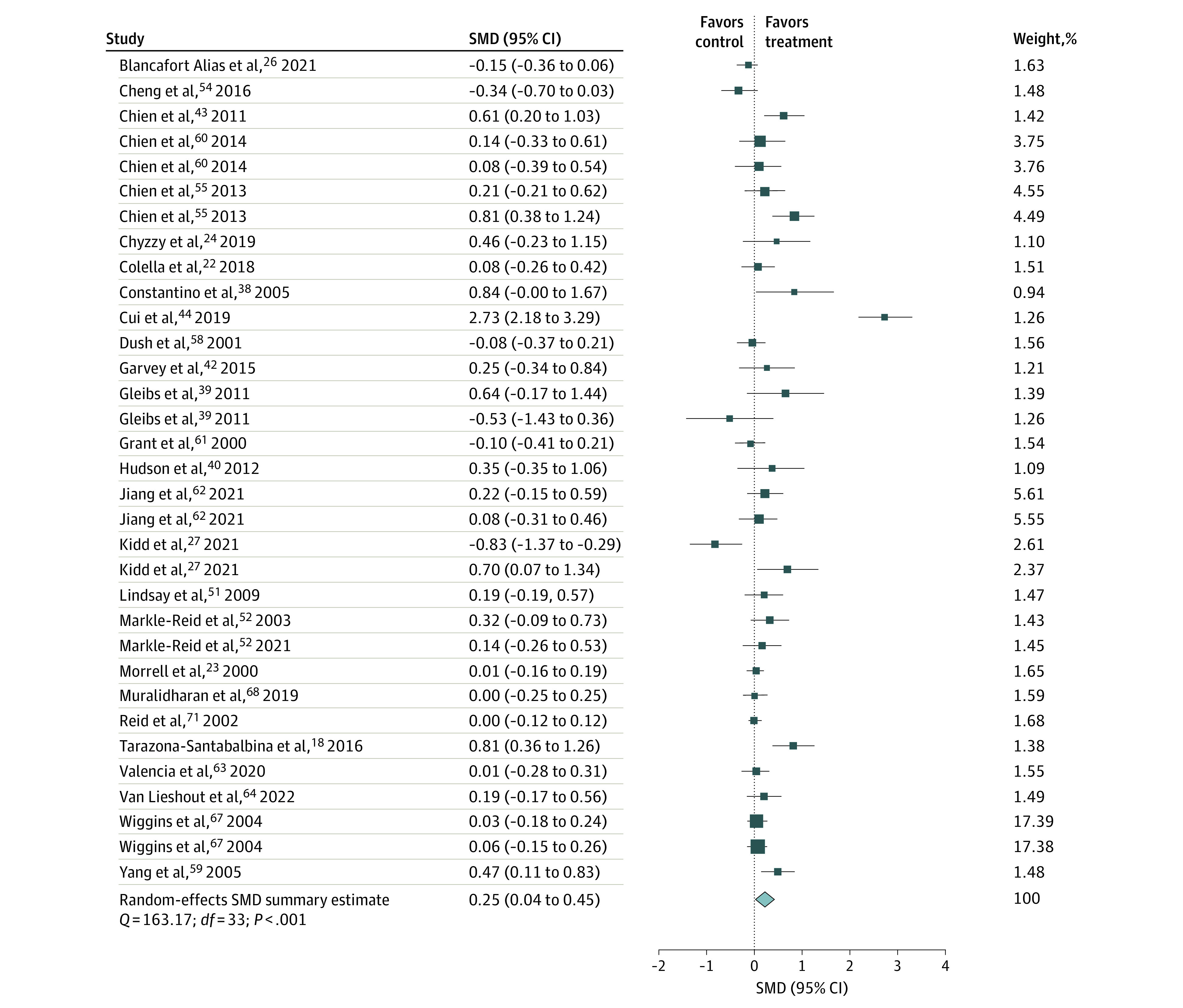
Pooled 95% CI Standardized Mean Difference of the Association of Psychosocial Interventions With Social Support Since multilevel meta-analyses included several effect estimates from each study, studies may be listed more than once for each analysis. Dots indicate estimates; whiskers, 95% CIs; diamond, summary estimate; SMD, standardized mean difference.

**Figure 5.**
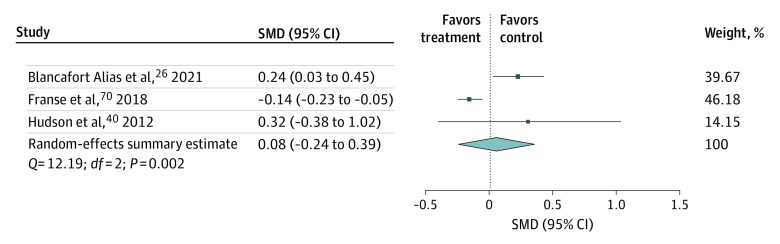
Pooled 95% CI Standardized Mean Difference of the Association of Psychosocial Interventions With Loneliness Since multilevel meta-analyses included several effect estimates from each study, studies may be listed more than once for each analysis. Dots indicate estimates; whiskers, 95% CIs; diamond, summary estimate; SMD, standardized mean difference.

Social support and loneliness were the primary domains of social well-being reported, and these were examined as secondary outcomes only in an SMD meta-analysis (Figure 4 and Figure 5). There was a statistically significant postintervention increase in social support but not in loneliness. Subgroup analyses of participants’ characteristics revealed a significant increase in social support following interventions conducted in China,^43,44,54,55,59,60^ among participants aged 30 to 60 years,^27,38,43,44,54,55,59,61,64,68^ men,^22,27,44,51,55,60,62,63,68^ and people with chronic illnesses.^22,42,44,51,62^ Analyses by intervention characteristics found improvements in social support following longer interventions, 1-on-1 interventions, and interventions delivered by health professionals.

### Assessment of Publication Bias

All the OR and SMD random-effects models were tested for publication bias, except for the model for loneliness due to the small number of studies (eFigure 2 in Supplement 1). No publication bias was found for the meta-analyses of ORs in either the immediate or sustained effect models. For the meta-analysis of SMD, the regression test for asymmetry reached statistical significance in both the immediate and sustained effect models, indicating significant publication bias. There was no bias found in the funnel plot of the SMD social support model (eFigure 2 in Supplement 1).

## Discussion

This systematic review and meta-analysis found that psychosocial interventions were associated with improved health care utilization. Models showed strong to moderate strength of evidence by BFs for number of inpatient visits and length of inpatients visits associated with social support. Emergency and primary care utilization also showed moderate strength of evidence for the OR model. Additionally, these interventions were associated with improvements in social support but not loneliness. Considering the need to reduce unnecessary health care spending in many countries, our findings have highlighted the potential for psychosocial interventions to reduce health care utilization. The largest reduction in health care use occurred in inpatient visit numbers and length of stay, emergency admissions, and primary care services. The reduction in inpatient and emergency care utilization could be due to an instrumental support system that may have prevented a health condition from deteriorating^73^ by direct care or by encouraging patients to seek treatment earlier.^14^ In addition, the support provided could also prevent or lower stress and anxiety associated with different health conditions,^74^ which may have contributed to reduction in unnecessary visits to the hospital.^75,76^ The decrease in health care use could also be a result of an improvement in overall health.^77,78^ These findings suggest that the emotional, informational, and instrumental support provided by psychosocial interventions may decrease the need to seek medical care excessively and may contribute to reduced health care expenses and improved service efficiency.^52,79,80,81^ Such outcomes may last over longer periods, although the current evidence on sustained outcomes is limited by the small number of studies with sustained follow-up.

Outpatient care was the only outcome that showed an increase. This supports previous findings of a positive association between social well-being and outpatient visits.^10,82^ Outpatient care often supports tertiary-level prevention, which can sustain recovery and reduce the need for urgent care.^83^ Therefore, our findings suggest that psychosocial interventions may lead to improved preventive care in the outpatient context, which in turn, may lead to less emergency and inpatient care utilization.^84,85^

Our subgroup analysis found a significant decrease in health care use among caregivers, suggesting the importance of including caregivers in psychosocial interventions. A significant decrease in health care use was also found among people with mental illness. As people with mental illnesses can experience poor social well-being due to isolation and perceived stigma,^86,87,88^ psychosocial interventions may provide much-needed social support for this group. Our subgroup analysis also found that interventions conducted in China and the US showed significant decreases in postintervention health care use, possibly due to differences in measurements or sample sizes.^89^ However, some of these differences may reflect country-specific variations in standards of care, service availability, health professional capacity, and cultural differences in health-seeking practices.^90,91,92^ We found that in interventions delivered by health professionals, there was a significant decrease in postintervention health care use. Health care professionals are often perceived as trusted sources of information and support, which may have contributed to more favorable outcomes. This finding echoes previous observations regarding implementing social care within health care settings^93,94^ and including health professionals in the delivery of psychosocial interventions.^85^ Finally, the decrease in health care use was more pronounced in individual-based interventions that included 1-on-1 activities. This finding highlights the importance of tailoring psychosocial interventions according to patients’ needs, as applied in case and disease management models.^85,95^

Our analysis also found a significant increase in postintervention social support. Previous studies found that loneliness and low social support were associated with more health care utilization,^10,96,97^ although a rapid review did not find a consistent association between psychosocial interventions and improvement in loneliness.^13^ Likewise, our study found no association of psychosocial interventions with loneliness, which is most likely due to the inconsistency in the results and the lack of power. These findings highlight the complex and measure-specific nature of the association between social well-being and health care utilization.^10^

### Limitations

This systematic review and meta-analysis has several limitations. First, the number of studies included in the analysis and the small sample sizes limited the certainty about the effect sizes and our ability to conduct multivariate analyses. Second, the use of ORs and SMDs is prone to measurement bias and therefore may have contributed to bias in the pooled effect size. Furthermore, most studies included in the SMD analysis reported means and SDs despite the underlying distributions being nonparametric. However, assuming that the SMD distribution shape remained the same, the SMD should provide a consistent estimate of the mean difference. In addition, synthesizing both ORs and SMDs should provide stronger and more complete evidence than relying on 1 measure of effect only. Third, the high level of heterogeneity among the studies may have contributed to the variability in the outcomes. We addressed this by conducting subgroup analyses. Fourth, the regression test for asymmetry indicated significant publication bias in the SMD meta-analysis, but since this analysis showed no association, the inclusion of unpublished data is unlikely to change our results. Fifth, relying on English abstracts may have led to the exclusion of eligible studies published in other languages.

## Conclusions

The findings of this systematic review and meta-analysis suggest that psychosocial interventions were associated with improvements in patients’ social support and health care use, possibly through increasing outpatient care while reducing the use of emergency and inpatient care. Our results also suggest that interventions should be tailored to the needs of patients and include health professionals as the deliverers. In addition, our findings about individual- and intervention-level characteristics may inform the design and implementation of future psychosocial interventions by providing information on the variables associated with intervention success. More randomized clinical trials with longer-term follow-ups are needed to better understand the sustained postintervention outcomes of health care use associated with psychosocial interventions.

## Notes

Supplement 1. **eTable 1.** Population, Intervention, Comparison, and Outcome (PICO) Table of Study Eligibility Criteria **eTable 2.** Study Search Strategy **eTable 3.** CINAHL Search Results **eTable 4.** Cochrane Search Results **eTable 5.** Embase Search Results **eTable 6.** Medline Search Results **eTable 7.** PsycInfo Search Results **eTable 8.** Scopus Search Results **eTable 9.** Summary Description of Randomized Clinical Trials Included in the Systematic Review (N = 41) **eTable 10.** Grading of Recommendations Assessment, Development and Evaluation (GRADE) Evidence Profile **eTable 11.** Strength of Evidence (Bayes Factors) in the OR and SMD According to Health Care Service **eTable 12.** Subgroup Analyses of Postintervention Health Care Utilization According to Participant- and Intervention-Level Factors **eFigure 1.** Cochrane Risk-of-Bias Summary and Author Judgments of Low, High and Unclear Risk of Bias Across All Included Studies (N = 41) **eFigure 2.** Publication Bias Funnel Plots for Standardized Mean Difference (SMD) and Log Odds Ratio (OR) Random Effect Models of Health Care Utilization Outcomes and Social Support **eFigure 3.** Pooled 95% CI Odds Ratio of the Sustained Association of Psychosocial Interventions With Health Care Utilization **eFigure 4.** Pooled 95% CI Standardized Mean Difference of the Sustained Association of Psychosocial Interventions With Health Care Utilization

Supplement 2.
Data Sharing Statement
