# Supplementary material for: Health Care Utilization Following Interventions to Improve Social Well-Being: A Systematic Review and Meta-analysis
Source: JAMA Netw Open. 2023 Jun 29;6(6):e2321019. doi: 10.1001/jamanetworkopen.2023.21019 (PMC10311391; doi:10.1001/jamanetworkopen.2023.21019)
Supplement: Supplement 1. — eTable 1. Population, Intervention, Comparison, and Outcome (PICO) Table of Study Eligibility Criteria eTable 2. Study Search Strategy eTable 3. CINAHL Search Results eTable 4. Cochrane Search Results eTable 5. Embase Search Results eTable 6. Medline Search Results eTable 7. PsycInfo Search Results eTable 8. Scopus Search Results eTable 9. Summary Description of Randomized Clinical Trials Included in the Systematic Review (N = 41) eTable 10. Grading of Recommendations Assessment, Development and Evaluation (GRADE) Evidence Profile eTable 11. Strength of Evidence (Bayes Factors) in the OR and SMD According to Health Care Service eTable 12. Subgroup Analyses of Postintervention Health Care Utilization According to Participant- and Intervention-Level Factors eFigure 1. Cochrane Risk-of-Bias Summary and Author Judgments of Low, High and Unclear Risk of Bias Across All Included Studies (N = 41) eFigure 2. Publication Bias Funnel Plots for Standardized Mean Difference (SMD) and Log Odds Ratio (OR) Random Effect Models of Health Care Utilization Outcomes and Social Support eFigure 3. Pooled 95% CI Odds Ratio of the Sustained Association of Psychosocial Interventions With Health Care Utilization eFigure 4. Pooled 95% CI Standardized Mean Difference of the Sustained Association of Psychosocial Interventions With Health Care Utilization [file jamanetwopen-e2321019-s001.pdf]

Supplemental Online Content

HaGani N, Surkalim DL, Clare PJ, Merom D, Smith BJ, Ding D. Health care utilization following interventions to improve social well-being: a systematic review and meta-analysis. *JAMA Netw Open.* 2023;6(6):e2321019. doi:10.1001/jamanetworkopen.2023.21019

eTable 1. Population, Intervention, Comparison, and Outcome (PICO) Table of Study Eligibility Criteria

eTable 2. Study Search Strategy

eTable 3. CINAHL Search Results

eTable 4. Cochrane Search Results

eTable 5. Embase Search Results

eTable 6. Medline Search Results

eTable 7. PsycInfo Search Results

eTable 8. Scopus Search Results

eTable 9. Summary Description of Randomized Clinical Trials Included in the Systematic Review (N = 41)

eTable 10. Grading of Recommendations Assessment, Development and Evaluation (GRADE) Evidence Profile

eTable 11. Strength of Evidence (Bayes Factors) in the OR and SMD According to Health Care Service

eTable 12. Subgroup Analyses of Postintervention Health Care Utilization According to Participant- and Intervention-Level Factors

eFigure 1. Cochrane Risk-of-Bias Summary and Author Judgments of Low, High and Unclear Risk of Bias Across All Included Studies (N = 41)

eFigure 2. Publication Bias Funnel Plots for Standardized Mean Difference (SMD) and Log Odds Ratio (OR) Random Effect Models of Health Care Utilization Outcomes and Social Support

eFigure 3. Pooled 95% CI Odds Ratio of the Sustained Association of Psychosocial Interventions With Health Care Utilization

eFigure 4. Pooled 95% CI Standardized Mean Difference of the Sustained Association of Psychosocial Interventions With Health Care Utilization

This supplemental material has been provided by the authors to give readers additional

eTable 1. Population, Intervention, Comparison, and Outcome (PICO) Table of Study Eligibility Criteria

P	**Patient, population, problem**<br>Participants from all population groups including healthy as well as clinical populations of all ages and genders.
I	**Intervention, prognostic factor or exposure**<br>Studies that investigated the effect of psychosocial interventions on participants' health care utilisation patterns and on levels of social wellbeing using a Randomised Control Trial (RCT) design. Psychosocial interventions were defined as those who aim to improve social, using a range of techniques such as therapeutic individual/ group support, group activities, peer support, community integration and physical activity.
C	**Comparison**<br>Interventions were compared to either standard care, an alternate intervention (e.g., individual activities, group activity without the main therapeutic component, providing information and manuals, medications) or wait-listed control.
O	**Outcome**<br>The primary outcomes were utilisation of health care services such as primary care, emergency care, inpatient care/readmissions, and outpatient care. The secondary outcomes were social wellbeing measures such as social support, social participation, social relationships, community support, social integration and loneliness.

© 2023 HaGani N et al. *JAMA Network Open.*

eTable 2. Study Search Strategy

Health care utilisation	Social wellbeing	Psychosocial interventions
Healthcare OR "health care" OR "re-admission*" OR admission* OR "emergency department*" OR "emergency room" OR hospitalization* OR hospitalisation OR "health service*" OR "general practi*" OR "ambulatory care" OR "urgent care" OR "physician* visit*" OR "primary care"	lonel* OR "social* isolat*" OR "social* disconnect*" OR "social support" OR "social interaction*" OR "peer support" OR "family support" OR "social network*" OR "social relationship*" OR "emotional support" OR "social problem*" OR "social capital" OR psychosocial.	"support intervention*" OR "brief intervention*" OR "group intervention*" OR "group-based program*" OR "Care Program*" OR "support program*" OR "psychosocial intervention*" OR "Multicomponent intervention*" OR "Self-Help Group*" OR "Transitional Care" OR "transitional intervention*" OR "education* intervention*" OR "follow-up care" OR "peer delivered intervention*" OR "support group*" OR "liaison service*".

© 2023 HaGani N et al. *JAMA Network Open.*

eTable 3. CINAHL Search Results

Thursday, May 13, 2021 10:39:38 AM

#	Query	Limiters/Expanders	Last Run Via	Results
S48	S45 AND S46 AND S47	Limiters - Human<br>Expanders - Apply equivalent subjects<br>Search modes - Boolean/Phrase	Interface - EBSCOhost Research Databases<br>Search Screen - Advanced Search<br>Database - CINAHL Complete	1,896
S47	S29 OR S30 OR S31 OR S32 OR S33 OR S34 OR S35 OR S36 OR S37 OR S38 OR S39 OR S40 OR S41 OR S42 OR S43 OR S44	Expanders - Apply equivalent subjects<br>Search modes - Boolean/Phrase	Interface - EBSCOhost Research Databases<br>Search Screen - Advanced Search<br>Database - CINAHL Complete	45,436
S46	S16 OR S17 OR S18 OR S19 OR S20 OR S21 OR S22 OR S23 OR S24 OR S25 OR S26 OR S27 OR S28	Expanders - Apply equivalent subjects<br>Search modes - Boolean/Phrase	Interface - EBSCOhost Research Databases<br>Search Screen - Advanced Search<br>Database - CINAHL Complete	175,402
S45	S1 OR S2 OR S3 OR S4 OR S5 OR S6 OR S7 OR S8 OR S9 OR S10 OR S11 OR S12 OR S13 OR S14 OR S15	Expanders - Apply equivalent subjects<br>Search modes - Boolean/Phrase	Interface - EBSCOhost Research Databases<br>Search Screen - Advanced Search<br>Database - CINAHL Complete	573,412
S44	AB "liaison service*"	Expanders - Apply equivalent subjects<br>Search modes - Boolean/Phrase	Interface - EBSCOhost Research Databases<br>Search Screen - Advanced Search<br>Database - CINAHL Complete	326
S43	(MH "Support Groups") OR AB "support group*"	Expanders - Apply equivalent subjects<br>Search modes - Boolean/Phrase	Interface - EBSCOhost Research Databases<br>Search Screen - Advanced Search<br>Database - CINAHL Complete	14,008
S42	AB "peer delivered intervention*"	Expanders - Apply equivalent subjects<br>Search modes - Boolean/Phrase	Interface - EBSCOhost Research Databases<br>Search Screen - Advanced Search<br>Database - CINAHL Complete	15

© 2023 HaGani N et al. *JAMA Network Open.*

S41	AB "follow up care"	Expanders - Apply equivalent subjects<br>Search modes - Boolean/Phrase	Interface - EBSCOhost Research Databases<br>Search Screen - Advanced Search<br>Database - CINAHL Complete	2,460
S40	AB "education* intervention*"	Expanders - Apply equivalent subjects<br>Search modes - Boolean/Phrase	Interface - EBSCOhost Research Databases<br>Search Screen - Advanced Search<br>Database - CINAHL Complete	8,544
S39	AB "transitional intervention*"	Expanders - Apply equivalent subjects<br>Search modes - Boolean/Phrase	Interface - EBSCOhost Research Databases<br>Search Screen - Advanced Search<br>Database - CINAHL Complete	19
S38	(MH "Transitional Care") OR AB "Transitional Care"	Expanders - Apply equivalent subjects<br>Search modes - Boolean/Phrase	Interface - EBSCOhost Research Databases<br>Search Screen - Advanced Search<br>Database - CINAHL Complete	2,795
S37	AB "Self Help Group*"	Expanders - Apply equivalent subjects<br>Search modes - Boolean/Phrase	Interface - EBSCOhost Research Databases<br>Search Screen - Advanced Search<br>Database - CINAHL Complete	742
S36	AB "Multicomponent intervention*"	Expanders - Apply equivalent subjects<br>Search modes - Boolean/Phrase	Interface - EBSCOhost Research Databases<br>Search Screen - Advanced Search<br>Database - CINAHL Complete	671
S35	(MH "Psychosocial Intervention") OR AB "psychosocial intervention*"	Expanders - Apply equivalent subjects<br>Search modes - Boolean/Phrase	Interface - EBSCOhost Research Databases<br>Search Screen - Advanced Search<br>Database - CINAHL Complete	3,011
S34	AB "support program*"	Expanders - Apply equivalent subjects<br>Search modes - Boolean/Phrase	Interface - EBSCOhost Research Databases<br>Search Screen - Advanced Search<br>Database - CINAHL Complete	2,461
S33	AB "Care Program*"	Expanders - Apply equivalent subjects<br>Search modes - Boolean/Phrase	Interface - EBSCOhost Research Databases<br>Search Screen - Advanced Search<br>Database - CINAHL Complete	5,650
S32	AB "group based program*"	Expanders - Apply equivalent subjects<br>Search modes - Boolean/Phrase	Interface - EBSCOhost Research Databases<br>Search Screen - Advanced Search<br>Database - CINAHL Complete	106

© 2023 HaGani N et al. *JAMA Network Open.*

S31	AB "group intervention*"	Expanders - Apply equivalent subjects<br>Search modes - Boolean/Phrase	Interface - EBSCOhost Research Databases<br>Search Screen - Advanced Search<br>Database - CINAHL Complete	2,635
S30	AB "brief intervention*"	Expanders - Apply equivalent subjects<br>Search modes - Boolean/Phrase	Interface - EBSCOhost Research Databases<br>Search Screen - Advanced Search<br>Database - CINAHL Complete	2,562
S29	AB "support intervention*"	Expanders - Apply equivalent subjects<br>Search modes - Boolean/Phrase	Interface - EBSCOhost Research Databases<br>Search Screen - Advanced Search<br>Database - CINAHL Complete	1,520
S28	(MH "Support, Psychosocial+") OR AB "psychosocial"	Expanders - Apply equivalent subjects<br>Search modes - Boolean/Phrase	Interface - EBSCOhost Research Databases<br>Search Screen - Advanced Search<br>Database - CINAHL Complete	129,174
S27	AB "social capital"	Expanders - Apply equivalent subjects<br>Search modes - Boolean/Phrase	Interface - EBSCOhost Research Databases<br>Search Screen - Advanced Search<br>Database - CINAHL Complete	2,587
S26	AB "social problem*"	Expanders - Apply equivalent subjects<br>Search modes - Boolean/Phrase	Interface - EBSCOhost Research Databases<br>Search Screen - Advanced Search<br>Database - CINAHL Complete	2,993
S25	AB "emotional support"	Expanders - Apply equivalent subjects<br>Search modes - Boolean/Phrase	Interface - EBSCOhost Research Databases<br>Search Screen - Advanced Search<br>Database - CINAHL Complete	4,978
S24	AB "social relationship*"	Expanders - Apply equivalent subjects<br>Search modes - Boolean/Phrase	Interface - EBSCOhost Research Databases<br>Search Screen - Advanced Search<br>Database - CINAHL Complete	3,224
S23	AB "social network*"	Expanders - Apply equivalent subjects<br>Search modes - Boolean/Phrase	Interface - EBSCOhost Research Databases<br>Search Screen - Advanced Search<br>Database - CINAHL Complete	10,678
S22	AB "family support"	Expanders - Apply equivalent subjects<br>Search modes - Boolean/Phrase	Interface - EBSCOhost Research Databases<br>Search Screen - Advanced Search<br>Database - CINAHL Complete	3,903
S21	AB "peer support"	Expanders - Apply equivalent subjects	Interface - EBSCOhost Research Databases	3,664

© 2023 HaGani N et al. *JAMA Network Open.*

		Search modes - Boolean/Phrase	Search Screen - Advanced Search<br>Database - CINAHL Complete	
S20	AB "social interaction*"	Expanders - Apply equivalent subjects<br>Search modes - Boolean/Phrase	Interface - EBSCOhost Research Databases<br>Search Screen - Advanced Search<br>Database - CINAHL Complete	6,396
S19	AB "social support"	Expanders - Apply equivalent subjects<br>Search modes - Boolean/Phrase	Interface - EBSCOhost Research Databases<br>Search Screen - Advanced Search<br>Database - CINAHL Complete	25,924
S18	AB "social* disconnect*"	Expanders - Apply equivalent subjects<br>Search modes - Boolean/Phrase	Interface - EBSCOhost Research Databases<br>Search Screen - Advanced Search<br>Database - CINAHL Complete	88
S17	(MH "Loneliness") OR AB "lonel*"	Expanders - Apply equivalent subjects<br>Search modes - Boolean/Phrase	Interface - EBSCOhost Research Databases<br>Search Screen - Advanced Search<br>Database - CINAHL Complete	7,248
S16	(MH "Social Isolation+") OR AB "social* isolat*"	Expanders - Apply equivalent subjects<br>Search modes - Boolean/Phrase	Interface - EBSCOhost Research Databases<br>Search Screen - Advanced Search<br>Database - CINAHL Complete	15,951
S15	(MH "Primary Health Care") OR AB "primary care"	Expanders - Apply equivalent subjects<br>Search modes - Boolean/Phrase	Interface - EBSCOhost Research Databases<br>Search Screen - Advanced Search<br>Database - CINAHL Complete	99,946
S14	AB "physician* visit*"	Expanders - Apply equivalent subjects<br>Search modes - Boolean/Phrase	Interface - EBSCOhost Research Databases<br>Search Screen - Advanced Search<br>Database - CINAHL Complete	1,527
S13	AB "urgent care"	Expanders - Apply equivalent subjects<br>Search modes - Boolean/Phrase	Interface - EBSCOhost Research Databases<br>Search Screen - Advanced Search<br>Database - CINAHL Complete	1,437
S12	AB "ambulatory care"	Expanders - Apply equivalent subjects<br>Search modes - Boolean/Phrase	Interface - EBSCOhost Research Databases<br>Search Screen - Advanced Search<br>Database - CINAHL Complete	3,218
S11	AB "general practi*"	Expanders - Apply equivalent subjects<br>Search modes - Boolean/Phrase	Interface - EBSCOhost Research Databases<br>Search Screen - Advanced Search<br>Database - CINAHL Complete	26,807

© 2023 HaGani N et al. *JAMA Network Open.*

S10	(MH "Health Services")	Expanders - Apply equivalent subjects Search modes - Boolean/Phrase	Interface - EBSCOhost Research Databases Search Screen - Advanced Search Database - CINAHL Complete	14,086
S9	AB "health service*"	Expanders - Apply equivalent subjects Search modes - Boolean/Phrase	Interface - EBSCOhost Research Databases Search Screen - Advanced Search Database - CINAHL Complete	60,164
S8	AB "hospitalisation*"	Expanders - Apply equivalent subjects Search modes - Boolean/Phrase	Interface - EBSCOhost Research Databases Search Screen - Advanced Search Database - CINAHL Complete	6,106
S7	AB "hospitalization*"	Expanders - Apply equivalent subjects Search modes - Boolean/Phrase	Interface - EBSCOhost Research Databases Search Screen - Advanced Search Database - CINAHL Complete	50,252
S6	AB "emergency room"	Expanders - Apply equivalent subjects Search modes - Boolean/Phrase	Interface - EBSCOhost Research Databases Search Screen - Advanced Search Database - CINAHL Complete	5,548
S5	AB "emergency department*"	Expanders - Apply equivalent subjects Search modes - Boolean/Phrase	Interface - EBSCOhost Research Databases Search Screen - Advanced Search Database - CINAHL Complete	47,815
S4	AB "admission*"	Expanders - Apply equivalent subjects Search modes - Boolean/Phrase	Interface - EBSCOhost Research Databases Search Screen - Advanced Search Database - CINAHL Complete	77,407
S3	AB "re-admission*"	Expanders - Apply equivalent subjects Search modes - Boolean/Phrase	Interface - EBSCOhost Research Databases Search Screen - Advanced Search Database - CINAHL Complete	899
S2	AB "healthcare"	Expanders - Apply equivalent subjects Search modes - Boolean/Phrase	Interface - EBSCOhost Research Databases Search Screen - Advanced Search Database - CINAHL Complete	135,531
S1	AB "health care"	Expanders - Apply equivalent subjects Search modes - Boolean/Phrase	Interface - EBSCOhost Research Databases Search Screen - Advanced Search Database - CINAHL Complete	176,976

Bottom of Form

eTable 4. Cochrane Search Results

Search Name: 13-5-21 search

Date Run: 13/05/2021 15:54:17

ID	Search Hits
#1	(healthcare):ti,ab,kw in Cochrane Reviews, Trials 21555
#2	("health care"):ti,ab,kw in Cochrane Reviews, Trials60101
#3	("re-admission*"):ti,ab,kw (Word variations have been searched) in Cochrane Reviews, Trials 771
#4	(admission*):ti,ab,kw (Word variations have been searched) in Cochrane Reviews, Trials 31651
#5	("emergency department*"):ti,ab,kw (Word variations have been searched) in Cochrane Reviews, Trials 10945
#6	("emergency room*"):ti,ab,kw in Cochrane Reviews, Trials 2109
#7	(hospitalization*):ti,ab,kw (Word variations have been searched) in Cochrane Reviews, Trials 40791
#8	("health service*"):ti,ab,kw (Word variations have been searched) in Cochrane Reviews, Trials 20082
#9	("general practice"):ti,ab,kw (Word variations have been searched) in Cochrane Reviews, Trials 6323
#10	("general practitioner"):ti,ab,kw (Word variations have been searched) in Cochrane Reviews, Trials 7274
#11	("ambulatory care"):ti,ab,kw in Cochrane Reviews, Trials 4809
#12	("urgent care"):ti,ab,kw in Cochrane Reviews, Trials 346
#13	("physician* visit*"):ti,ab,kw (Word variations have been searched) in Cochrane Reviews, Trials 531
#14	MeSH descriptor: [Health Services] this term only 452
#15	("primary care"):ti,ab,kw in Cochrane Reviews, Trials 18852
#16	MeSH descriptor: [Physicians] explode all trees 2093
#17	MeSH descriptor: [Primary Health Care] explode all trees 7508
#18	MeSH descriptor: [Social Isolation] explode all trees 301

© 2023 HaGani N et al. *JAMA Network Open.*

#19 (social isolation):ti,ab,kw (Word variations have been searched) in Cochrane Reviews, Trials 1568
#20 (lonel*):ti,ab,kw (Word variations have been searched) in Cochrane Reviews, Trials 780
#21 MeSH descriptor: [Social Isolation] explode all trees 301
#22 ("social support"):ti,ab,kw (Word variations have been searched) in Cochrane Reviews, Trials 8345
#23 ("social interaction*"):ti,ab,kw (Word variations have been searched) in Cochrane Reviews, Trials 3576
#24 ("peer support"):ti,ab,kw (Word variations have been searched) in Cochrane Reviews, Trials 1277
#25 ("family support"):ti,ab,kw in Cochrane Reviews, Trials 586
#26 ("social network*"):ti,ab,kw (Word variations have been searched) in Cochrane Reviews, Trials 1665
#27 ("social relationship*"):ti,ab,kw (Word variations have been searched) in Cochrane Reviews, Trials 499
#28 ("emotional support"):ti,ab,kw in Cochrane Reviews, Trials 624
#29 ("social problems*"):ti,ab,kw in Cochrane Reviews, Trials 350
#30 (psychosocial):ti,ab,kw in Cochrane Reviews, Trials 16174
#31 ("social capital"):ti,ab,kw in Cochrane Reviews, Trials 122
#32 ("support intervention*"):ti,ab,kw (Word variations have been searched) in Cochrane Reviews, Trials 1617
#33 ("brief intervention*"):ti,ab,kw in Cochrane Reviews, Trials 2077
#34 ("group intervention*"):ti,ab,kw in Cochrane Reviews, Trials 5057
#35 ("group based program*"):ti,ab,kw in Cochrane Reviews, Trials 79
#36 ("Care Program*"):ti,ab,kw in Cochrane Reviews, Trials 1863
#37 ("support program*"):ti,ab,kw (Word variations have been searched) in Cochrane Reviews, Trials 1241
#38 ("psychosocial intervention*"):ti,ab,kw in Cochrane Reviews, Trials 1193
#39 MeSH descriptor: [Psychosocial Intervention] explode all trees 23

© 2023 HaGani N et al. *JAMA Network Open.*

#40 ("Multicomponent intervention*"):ti,ab,kw (Word variations have been searched) in Cochrane Reviews, Trials 877	
#41 ("Self-Help Group*"):ti,ab,kw (Word variations have been searched) in Cochrane Reviews, Trials 908	
#42 ("transitional intervention*"):ti,ab,kw in Cochrane Reviews, Trials 12	
#43 ("education intervention*"):ti,ab,kw (Word variations have been searched) in Cochrane Reviews, Trials 6480	
#44 ("follow-up care"):ti,ab,kw in Cochrane Reviews, Trials 706	
#45 ("peer delivered intervention*"):ti,ab,kw in Cochrane Reviews, Trials 15	
#46 ("support group*"):ti,ab,kw (Word variations have been searched) in Cochrane Reviews, Trials 2111	
#47 ("liaison service*"):ti,ab,kw in Cochrane Reviews, Trials 49	
#48 #1 #2 or #3 or #4 or #5 or #6 #7 or #8 or #9 or #10 or #11 or #12 or #13 or #14 or #15 or #16 or #17 94297	
#49 #18 or #19 or #20 or #21 or #22 or #23 or #24 or #25 or #26 or #27 or #28 or #29 or #30 or #31 30568	
#50 #32 or #33 or # 35 or #35 or #36 or #37 or #38 or #39 or #40 or #41 or #42 or #43 or #44 or #45 or #46 or #47 162590	
#51 #48 and #49 and #50 1321	
#52 ("Psychosocial Group*"):ti,ab,kw in Cochrane Reviews, Trials 96	
#53 #50 or #52 162659	
#54 #48 and #49 and #53 1328	

Search Name: Cochrane update

Last Saved: 01/12/2022 11:04:40

Comment:

ID	Search
#1	(healthcare):ti,ab,kw
#2	("health care"):ti,ab,kw
#3	("re-admission*"):ti,ab,kw (Word variations have been searched)
#4	(admission*):ti,ab,kw (Word variations have been searched)

© 2023 HaGani N et al. *JAMA Network Open.*

#5	("emergency department*"):ti,ab,kw (Word variations have been searched)
#6	("emergency room*"):ti,ab,kw
#7	(hospitalization*):ti,ab,kw (Word variations have been searched)
#8	("health service*"):ti,ab,kw (Word variations have been searched)
#9	("general practice"):ti,ab,kw (Word variations have been searched)
#10	("general practitioner"):ti,ab,kw (Word variations have been searched)
#11	("ambulatory care"):ti,ab,kw
#12	("urgent care"):ti,ab,kw
#13	("physician* visit*"):ti,ab,kw (Word variations have been searched)
#14	MeSH descriptor: [Health Services] this term only
#15	("primary care"):ti,ab,kw
#16	MeSH descriptor: [Physicians] explode all trees
#17	MeSH descriptor: [Primary Health Care] explode all trees
#18	MeSH descriptor: [Social Isolation] explode all trees
#19	(social isolation):ti,ab,kw (Word variations have been searched)
#20	(lonel*):ti,ab,kw (Word variations have been searched)
#21	("social support"):ti,ab,kw (Word variations have been searched)
#22	("social interaction*"):ti,ab,kw (Word variations have been searched)
#23	("peer support"):ti,ab,kw (Word variations have been searched)
#24	("family support"):ti,ab,kw
#25	("social network*"):ti,ab,kw (Word variations have been searched)
#26	("social relationship*"):ti,ab,kw (Word variations have been searched)
#27	("emotional support"):ti,ab,kw
#28	("social problems*"):ti,ab,kw
#29	(psychosocial):ti,ab,kw
#30	("social capital"):ti,ab,kw
#31	("support intervention*"):ti,ab,kw (Word variations have been searched)
#32	("brief intervention*"):ti,ab,kw
#33	("group intervention*"):ti,ab,kw

© 2023 HaGani N et al. *JAMA Network Open.*

#34	("group based program*"):ti,ab,kw
#35	("Care Program*"):ti,ab,kw
#36	("support program*"):ti,ab,kw (Word variations have been searched)
#37	("psychosocial intervention*"):ti,ab,kw
#38	MeSH descriptor: [Psychosocial Intervention] explode all trees
#39	("Multicomponent intervention*"):ti,ab,kw (Word variations have been searched)
#40	("Self-Help Group*"):ti,ab,kw (Word variations have been searched)
#41	("transitional intervention*"):ti,ab,kw
#42	("education intervention*"):ti,ab,kw (Word variations have been searched)
#43	("follow-up care"):ti,ab,kw
#44	("peer delivered intervention*"):ti,ab,kw
#45	("support group*"):ti,ab,kw
#46	("liaison service*"):ti,ab,kw
#47	("Psychosocial Group*"):ti,ab,kw
#48	#1 #2 or #3 or #4 or #5 or #6 #7 or #8 or #9 or #10 or #11 or #12 or #13 or #14 or #15 or #16 or #17
#49	#18 or #19 or #20 or #21 or #22 or #23 or #24 or #25 or #26 or #27 or #28 or #29 or #30
#50	#31 or #32 or #33 or # 35 or #35 or #36 or #37 or #38 or #39 or #40 or #41 or #42 or #43 or #44 or #45 or #46 or #47
#51	#48 and #49 and #50 with Cochrane Library publication date Between May 2021 and Dec 2022, in Cochrane Reviews, Trials

eTable 5. Embase Search Results

Database: Embase Classic <1947 to 1973>, Embase <1974 to 2021 May 12>
Search Strategy:

1	healthcare.ti,ab. (387663)
2	"health care".ti,ab. (465341)
3	"re-admission*".ti,ab. (5862)
4	admission*.ti,ab. (422158)
5	"emergency department*".ti,ab. (155466)
6	"emergency room".ti,ab. (34498)
7	hospitali#ation*.ti,ab. (300864)
8	"health service*".ti,ab. (143650)
9	"general practi*".ti,ab. (109279)
10	"ambulatory care".ti,ab. (12607)
11	"urgent care".ti,ab. (3984)
12	"physician* visit*".ti,ab. or Health Services/ (167143)
13	Social Isolation/ or Loneliness/ or lonel*.ti,ab. (37906)
14	"social* isolat*".ti,ab. (12534)
15	"social* disconnect*".ti,ab. (198)
16	"social support".ti,ab. or Social Support/ (109555)
17	"social interaction*".ti,ab. (27181)
18	"peer support".ti,ab. (6943)
19	"family support".ti,ab. (8024)
20	"social network*".ti,ab. (22566)
21	"social relationship*".ti,ab. (8832)
22	"emotional support".ti,ab. (9032)
23	"social problem*".ti,ab. (11632)
24	"support intervention*".ti,ab. (3323)
25	"brief intervention*".ti,ab. (5691)
26	"group intervention*".ti,ab. (6208)
27	"social capital".ti,ab. (4155)
28	"group based program*".ti,ab. (218)
29	"Care Program*".ti,ab. (16651)
30	(support adj2 program*).ti,ab. (10650)
31	"primary care physician".ti,ab. or Physicians, Primary Care/ (114259)
32	psychosocial.ti,ab. (138715)
33	"psychosocial intervention".ti,ab. or Psychosocial Intervention/ (2936)
34	13 or 14 or 15 or 16 or 17 or 18 or 19 or 20 or 21 or 22 or 23 or 27 or 32 (341682)
35	(Multicomponent adj2 intervention*).ti,ab. (1648)

© 2023 HaGani N et al. *JAMA Network Open.*

36	"Self-Help Group*".ti,ab. (2364)
37	Transitional Care/ or "transitional intervention*".ti,ab. (3688)
38	"education intervention*".ti,ab. (4518)
39	24 or 25 or 26 or 28 or 29 or 30 or 33 or 35 or 36 or 37 or 38 (56471)
40	"follow-up care".ti,ab. (7727)
41	39 or 40 (63969)
42	"peer delivered intervention*".ti,ab. (39)
43	41 or 42 (64001)
44	Support Groups/ or "support group*".ti,ab. (12173)
45	43 or 44 (74959)
46	"liaison service*".ti,ab. (1832)
47	45 or 46 (76747)
48	1 or 2 or 3 or 4 or 5 or 6 or 7 or 8 or 9 or 10 or 11 or 12 or 31 (1867401)
49	34 and 47 and 48 (4359)
50	limit 49 to humans (4108)

Embase Classic <1947 to 1973>
Embase <1974 to 2022 November 30>

1	healthcare.ti,ab.	470347
2	"health care".ti,ab.	509070
3	"re-admission*".ti,ab.	6798
4	admission*.ti,ab.	481845
5	"emergency department*".ti,ab.	180175
6	"emergency room".ti,ab.	39117
7	hospitali#ation*.ti,ab.	351309
8	"health service*".ti,ab.	158935
9	"general practi*".ti,ab.	114689
10	"ambulatory care".ti,ab.	13614
11	"urgent care".ti,ab.	4857
12	"physician* visit*".ti,ab. or Health Services/	174748
13	Social Isolation/ or Loneliness/ or lonel*.ti,ab.	45202
14	"social* isolat*".ti,ab.	15912
15	"social* disconnect*".ti,ab.	288
16	"social support".ti,ab. or Social Support/	122087
17	"social interaction*".ti,ab.	31122
18	"peer support".ti,ab.	8627
19	"family support".ti,ab.	9219
20	"social network*".ti,ab.	26260
21	"social relationship*".ti,ab.	10052
22	"emotional support".ti,ab.	10458
23	"social problem*".ti,ab.	12378
24	"support intervention*".ti,ab.	3977
25	"brief intervention*".ti,ab.	6271
26	"group intervention*".ti,ab.	6901
27	"social capital".ti,ab.	4827
28	"group based program*".ti,ab.	252
29	"Care Program*".ti,ab.	18163

30	(support adj2 program*).ti,ab.	12456
31	"primary care physician".ti,ab. or Physicians, Primary Care/	122056
32	psychosocial.ti,ab.	153351
33	"psychosocial intervention".ti,ab. or Psychosocial Intervention/	4141
34	13 or 14 or 15 or 16 or 17 or 18 or 19 or 20 or 21 or 22 or 23 or 27 or 32	385235
35	(Multicomponent adj2 intervention*).ti,ab.	2051
36	"Self-Help Group*".ti,ab.	2439
37	Transitional Care/ or "transitional intervention*".ti,ab.	4881
38	"education intervention*".ti,ab.	5238
39	24 or 25 or 26 or 28 or 29 or 30 or 33 or 35 or 36 or 37 or 38	65042
40	"follow-up care".ti,ab.	8793
41	39 or 40	73573
42	"peer delivered intervention*".ti,ab.	47
43	41 or 42	73612
44	Support Groups/ or "support group*".ti,ab.	13507
45	43 or 44	85775
46	"liaison service*".ti,ab.	2113
47	45 or 46	87832
48	1 or 2 or 3 or 4 or 5 or 6 or 7 or 8 or 9 or 10 or 11 or 12 or 31	2105865
49	34 and 47 and 48	5258
50	limit 49 to (human and yr="2021 -Current")	1090

eTable 6. Medline Search Results

Database: Ovid MEDLINE(R) ALL <1946 to May 12, 2021>
Search Strategy:

1	healthcare.ti,ab. (241421)
2	"health care".ti,ab. (363393)
3	"re-admission*".ti,ab. (2298)
4	admission*.ti,ab. (236552)
5	"emergency department*".ti,ab. (101017)
6	"emergency room".ti,ab. (19077)
7	hospitali#ation*.ti,ab. (173689)
8	"health service*".ti,ab. (115631)
9	"general practi*".ti,ab. (83153)
10	"ambulatory care".ti,ab. (9393)
11	"urgent care".ti,ab. (2429)
12	"physician* visit*".ti,ab. or Health Services/ (28654)
13	Social Isolation/ or Loneliness/ or lonel*.ti,ab. (22920)
14	"social* isolat*".ti,ab. (9243)
15	"social* disconnect*".ti,ab. (162)
16	"social support".ti,ab. or Social Support/ (93356)
17	"social interaction*".ti,ab. (21694)
18	"peer support".ti,ab. (4838)
19	"family support".ti,ab. (5776)
20	"social network*".ti,ab. (18841)
21	"social relationship*".ti,ab. (6783)
22	"emotional support".ti,ab. (6566)
23	"social problem*".ti,ab. (8366)
24	"support intervention*".ti,ab. (2545)
25	"brief intervention*".ti,ab. (4232)
26	"group intervention*".ti,ab. (3594)
27	"social capital".ti,ab. (3879)
28	"group based program*".ti,ab. (156)
29	"Care Program*".ti,ab. (12258)
30	(support adj2 program*).ti,ab. (7672)
31	"primary care physician".ti,ab. or Physicians, Primary Care/ (9596)
32	psychosocial.ti,ab. (100299)
33	"psychosocial intervention".ti,ab. or Psychosocial Intervention/ (2008)
34	13 or 14 or 15 or 16 or 17 or 18 or 19 or 20 or 21 or 22 or 23 or 27 or 32 (258905)
35	(Multicomponent adj2 intervention*).ti,ab. (1488)

© 2023 HaGani N et al. *JAMA Network Open.*

36	"Self-Help Group*".ti,ab. (1705)
37	Transitional Care/ or "transitional intervention*".ti,ab. (927)
38	"education intervention*".ti,ab. (3347)
39	24 or 25 or 26 or 28 or 29 or 30 or 33 or 35 or 36 or 37 or 38 (39025)
40	"follow-up care".ti,ab. (4980)
41	39 or 40 (43882)
42	"peer delivered intervention*".ti,ab. (37)
43	41 or 42 (43915)
44	Support Groups/ or "support group*".ti,ab. (14930)
45	43 or 44 (56883)
46	"liaison service*".ti,ab. (963)
47	45 or 46 (57823)
48	1 or 2 or 3 or 4 or 5 or 6 or 7 or 8 or 9 or 10 or 11 or 12 or 31 (1169975)
49	34 and 47 and 48 (2850)
50	limit 49 to humans (2392)

Ovid MEDLINE(R) ALL <1946 to November 29, 2022>

1	healthcare.ti,ab.	303752
2	"health care".ti,ab.	402595
3	"re-admission*".ti,ab.	2665
4	admission*.ti,ab.	271514
5	"emergency department*".ti,ab.	117922
6	"emergency room".ti,ab.	21412
7	hospitali#ation*.ti,ab.	205033
8	"health service*".ti,ab.	129155
9	"general practi*".ti,ab.	88399
10	"ambulatory care".ti,ab.	10125
11	"urgent care".ti,ab.	2945
12	"physician* visit*".ti,ab. or Health Services/	30664
13	Social Isolation/ or Loneliness/ or lonel*.ti,ab.	27179
14	"social* isolat*".ti,ab.	11993
15	"social* disconnect*".ti,ab.	249
16	"social support".ti,ab. or Social Support/	102293
17	"social interaction*".ti,ab.	25531
18	"peer support".ti,ab.	6175
19	"family support".ti,ab.	6790
20	"social network*".ti,ab.	22721
21	"social relationship*".ti,ab.	8031
22	"emotional support".ti,ab.	7783
23	"social problem*".ti,ab.	9125
24	"support intervention*".ti,ab.	3124
25	"brief intervention*".ti,ab.	4722
26	"group intervention*".ti,ab.	4148
27	"social capital".ti,ab.	4717
28	"group based program*".ti,ab.	189
29	"Care Program*".ti,ab.	13476

30	(support adj2 program*).ti,ab.	9147
31	"primary care physician".ti,ab. or Physicians, Primary Care/	10476
32	psychosocial.ti,ab.	112801
33	"psychosocial intervention".ti,ab. or Psychosocial Intervention/	2817
34	13 or 14 or 15 or 16 or 17 or 18 or 19 or 20 or 21 or 22 or 23 or 27 or 32	294963
35	(Multicomponent adj2 intervention*).ti,ab.	1869
36	"Self-Help Group*".ti,ab.	1810
37	Transitional Care/ or "transitional intervention*".ti,ab.	1221
38	"education intervention*".ti,ab.	3949
39	24 or 25 or 26 or 28 or 29 or 30 or 33 or 35 or 36 or 37 or 38	45340
40	"follow-up care".ti,ab.	5708
41	39 or 40	50907
42	"peer delivered intervention*".ti,ab.	44
43	41 or 42	50945
44	Support Groups/ or "support group*".ti,ab.	15873
45	43 or 44	64731
46	"liaison service*".ti,ab.	1090
47	45 or 46	65794
48	1 or 2 or 3 or 4 or 5 or 6 or 7 or 8 or 9 or 10 or 11 or 12 or 31	1342007
49	34 and 47 and 48	3416
50	limit 49 to (human and yr="2021 -Current")	512

© 2023 HaGani N et al. *JAMA Network Open.*

eTable 7. PsycInfo Search Results

Database: APA PsycInfo <1806 to May Week 1 2021>
Search Strategy:

1	healthcare.ti,ab. (47592)
2	"health care".ti,ab. (98809)
3	"re-admission*".ti,ab. (235)
4	admission*.ti,ab. (38649)
5	"emergency department*".ti,ab. (9255)
6	"emergency room".ti,ab. (3392)
7	hospitali#ation*.ti,ab. (27965)
8	"health service*".ti,ab. (48648)
9	"general practi*".ti,ab. (13209)
10	"ambulatory care".ti,ab. (1109)
11	"urgent care".ti,ab. (319)
12	"physician* visit*".ti,ab. or Health Services/ (790)
13	Social Isolation/ or Loneliness/ or lonel*.ti,ab. (18839)
14	"social* isolat*".ti,ab. (8435)
15	"social* disconnect*".ti,ab. (207)
16	"social support".ti,ab. or Social Support/ (62943)
17	"social interaction*".ti,ab. (31787)
18	"peer support".ti,ab. (4480)
19	"family support".ti,ab. (6519)
20	"social network*".ti,ab. (26040)
21	"social relationship*".ti,ab. (11856)
22	"emotional support".ti,ab. (7089)
23	"social problem*".ti,ab. (10998)
24	"support intervention*".ti,ab. (1474)
25	"brief intervention*".ti,ab. (3619)
26	"group intervention*".ti,ab. (4536)
27	"social capital".ti,ab. (8090)
28	"group based program*".ti,ab. (106)
29	"Care Program*".ti,ab. (4112)
30	(support adj2 program*).ti,ab. (6000)
31	"primary care physician".ti,ab. or Physicians, Primary Care/ (1234)
32	psychosocial.ti,ab. (83959)
33	"psychosocial intervention".ti,ab. or Psychosocial Intervention/ (1815)
34	13 or 14 or 15 or 16 or 17 or 18 or 19 or 20 or 21 or 22 or 23 or 27 or 32 (244612)
35	(Multicomponent adj2 intervention*).ti,ab. (741)

© 2023 HaGani N et al. *JAMA Network Open.*

36	"Self-Help Group*".ti,ab. (1895)
37	Transitional Care/ or "transitional intervention*".ti,ab. (22)
38	"education intervention*".ti,ab. (1806)
39	24 or 25 or 26 or 28 or 29 or 30 or 33 or 35 or 36 or 37 or 38 (25542)
40	"follow-up care".ti,ab. (1109)
41	39 or 40 (26608)
42	"peer delivered intervention*".ti,ab. (29)
43	41 or 42 (26635)
44	Support Groups/ or "support group*".ti,ab. (9597)
45	43 or 44 (34866)
46	"liaison service*".ti,ab. (556)
47	45 or 46 (35411)
48	1 or 2 or 3 or 4 or 5 or 6 or 7 or 8 or 9 or 10 or 11 or 12 or 31 (248274)
49	34 and 47 and 48 (1433)
50	limit 49 to human (1374)

APA PsycInfo <1806 to November Week 3 2022>

1	healthcare.ti,ab.	57679
2	"health care".ti,ab.	107254
3	"re-admission*".ti,ab.	257
4	admission*.ti,ab.	41072
5	"emergency department*".ti,ab.	10507
6	"emergency room".ti,ab.	3616
7	hospitali#ation*.ti,ab.	30165
8	"health service*".ti,ab.	53487
9	"general practi*".ti,ab.	13822
10	"ambulatory care".ti,ab.	1176
11	"urgent care".ti,ab.	369
12	"physician* visit*".ti,ab. or Health Services/	830
13	Social Isolation/ or Loneliness/ or lonel*.ti,ab.	21631
14	"social* isolat*".ti,ab.	9886
15	"social* disconnect*".ti,ab.	277
16	"social support".ti,ab. or Social Support/	68983
17	"social interaction*".ti,ab.	34790
18	"peer support".ti,ab.	5270
19	"family support".ti,ab.	7134
20	"social network*".ti,ab.	28809
21	"social relationship*".ti,ab.	12912
22	"emotional support".ti,ab.	7898
23	"social problem*".ti,ab.	11579
24	"support intervention*".ti,ab.	1711
25	"brief intervention*".ti,ab.	3967
26	"group intervention*".ti,ab.	4950
27	"social capital".ti,ab.	8867
28	"group based program*".ti,ab.	129

29	"Care Program*".ti,ab.	4382
30	(support adj2 program*).ti,ab.	6725
31	"primary care physician".ti,ab. or Physicians, Primary Care/	1282
32	psychosocial.ti,ab.	90510
33	"psychosocial intervention".ti,ab. or Psychosocial Intervention/	1971
34	13 or 14 or 15 or 16 or 17 or 18 or 19 or 20 or 21 or 22 or 23 or 27 or 32	267169
35	(Multicomponent adj2 intervention*).ti,ab.	869
36	"Self-Help Group*".ti,ab.	1960
37	Transitional Care/ or "transitional intervention*".ti,ab.	23
38	"education intervention*".ti,ab.	1992
39	24 or 25 or 26 or 28 or 29 or 30 or 33 or 35 or 36 or 37 or 38	28030
40	"follow-up care".ti,ab.	1236
41	39 or 40	29219
42	"peer delivered intervention*".ti,ab.	34
43	41 or 42	29251
44	Support Groups/ or "support group*".ti,ab.	10106
45	43 or 44	37937
46	"liaison service*".ti,ab.	585
47	45 or 46	38510
48	1 or 2 or 3 or 4 or 5 or 6 or 7 or 8 or 9 or 10 or 11 or 12 or 31	273435
49	34 and 47 and 48	1643
50	limit 49 to (human and yr="2021 -Current")	149

eTable 8. Scopus Search Results

ID	Name	Query	Documents	Date last run
result#7	title search	(TITLE (healthcare OR "health care" OR "re-admission*" OR admission* OR "emergency department*" OR "emergency room" OR hospitalization* OR hospitalisation OR "health service*" OR "general practi*" OR "ambulatory care" OR "urgent care" OR "physician* visit*" OR "primary care") AND TITLE (lonel* OR "social* isolat*" OR "social* disconnect*" OR "social support" OR "social interaction*" OR "peer support" OR "family support" OR "social network*" OR "social relationship*" OR "emotional support" OR "social problem*" OR "social capital" OR psychosocial) AND TITLE ("support intervention*" OR "brief intervention*" OR "group intervention*" OR "group-based program*" OR "Care Program*" OR "support program*" OR "psychosocial intervention*" OR "Multicomponent intervention*" OR "Self-Help Group*" OR "Transitional Care" OR "transitional intervention*" OR "education* intervention*" OR "follow-up care" OR "peer delivered intervention*" OR "support group*" OR "liaison service*"))<br>View Less_<br>_Edit query	71	17 May 2021_
result#6	AB search any year-2013	(ABS (healthcare OR "health care" OR "re-admission*" OR admission* OR "emergency department*" OR "emergency room" OR hospitalization* OR hospitalisation OR "health service*" OR "general practi*" OR "ambulatory care" OR "urgent care" OR "physician* visit*" OR "primary care") AND ABS (lonel* OR "social* isolat*" OR "social* disconnect*" OR "social support" OR "social interaction*" OR "peer support" OR "family support" OR "social network*" OR "social relationship*" OR "emotional support" OR "social problem*" OR "social capital" OR psychosocial) AND ABS ("support intervention*" OR "brief intervention*" OR "group intervention*" OR "group-based program*" OR "Care Program*" OR "support program*" OR "psychosocial intervention*" OR "Multicomponent intervention*" OR "Self-Help Group*" OR "Transitional Care" OR "transitional intervention*" OR "education* intervention*" OR "follow-up care" OR "peer delivered intervention*" OR "support group*" OR "liaison service*")) AND PUBYEAR < 2014<br>View Less_<br>_Edit query	1,695	17 May 2021_
result#3	AB search 2014-today	(ABS (healthcare OR "health care" OR "re-admission*" OR admission* OR "emergency department*" OR "emergency room" OR hospitalization* OR hospitali	1,758	17 May 2021_

© 2023 HaGani N et al. *JAMA Network Open.*

ID	Name	Query	Documents	Date last run
		sation OR "health service*" OR "general practi*" OR "ambulatory care" OR "urgent care" OR "physician* visit*" OR "primary care") AND ABS (lonel* OR "social* isolat*" OR "social* disconnect*" OR "social support" OR "social interaction*" OR "peer support" OR "family support" OR "social network*" OR "social relationship*" OR "emotional support" OR "social problem*" OR "social capital" OR psychosocial) AND ABS ("support intervention*" OR "brief intervention*" OR "group intervention*" OR "group-based program*" OR "Care Program*" OR "support program*" OR "psychosocial intervention*" OR "Multicomponent intervention*" OR "Self-Help Group*" OR "Transitional Care" OR "transitional intervention*" OR "education* intervention*" OR "follow-up care" OR "peer delivered intervention*" OR "support group*" OR "liaison service*")) AND PUBYEAR > 2013 AND PUBYEAR < 2022<br>View Less_<br>_Edit query		
result#2	search ti-ab-kw	(TITLE-ABS-KEY (healthcare OR "health care" OR "re-admission*" OR admission* OR "emergency department*" OR "emergency room" OR hospitalization* OR hospitalisation OR "health service*" OR "general practi*" OR "ambulatory care" OR "urgent care" OR "physician* visit*" OR "primary care") AND TITLE-ABS-KEY (lonel* OR "social* isolat*" OR "social* disconnect*" OR "social support" OR "social interaction*" OR "peer support" OR "family support" OR "social network*" OR "social relationship*" OR "emotional support" OR "social problem*" OR "social capital" OR psychosocial) AND TITLE-ABS-KEY ("support intervention*" OR "brief intervention*" OR "group intervention*" OR "group-based program*" OR "Care Program*" OR "support program*" OR "psychosocial intervention*" OR "Multicomponent intervention*" OR "Self-Help Group*" OR "Transitional Care" OR "transitional intervention*" OR "education* intervention*" OR "follow-up care" OR "peer delivered intervention*" OR "support group*" OR "liaison service*")) AND (LIMIT-TO (SUBJAREA , "MEDI") OR LIMIT-TO (SUBJAREA , "NURS") OR LIMIT-TO (SUBJAREA , "PSYC") OR LIMIT-TO (SUBJAREA , "SOCI")) ... View More_	10,022	17 May 2021_

© 2023 HaGani N et al. *JAMA Network Open.*

Combine queries...*e.g. #1 AND NOT #3*

ID	Name	Query	Documents	Date last run	Actions
result#1	scopus update	(TITLE-ABS-KEY (healthcare OR "health care" OR "re-admission*" OR admission* OR "emergency department*" OR "emergency room" OR hospitalization* OR hospitalisation OR "health service*" OR "general practi*" OR "ambulatory care" OR "urgent care" OR "physician* visit*" OR "primary care") AND TITLE-ABS-KEY (lonel* OR "social* isolat*" OR "social* disconnect*" OR "social support" OR "social interaction*" OR "peer support" OR "family support" OR "social network*" OR "social relationship*" OR "emotional support" OR "social problem*" OR "social capital" OR psychosocial) AND TITLE-ABS-KEY ("support intervention*" OR "brief intervention*" OR "group intervention*" OR "group-based program*" OR "Care Program*" OR "support program*" OR "psychosocial intervention*" OR "Multicomponent intervention*" OR "Self-Help Group*" OR "Transitional Care" OR "transitional intervention*" OR "education* intervention*" OR "follow-up care" OR "peer delivered intervention*" OR "support group*" OR "liaison service*")) AND PUBYEAR > 2020<br>View Less<br>Edit query	2,003	01 Dec 2022	• Edit the search or change the name<br>• Create new saved search based on this search<br>•<br>• Set alert<br>• Delete saved search

© 2023 HaGani N et al. *JAMA Network Open.*

eTable 9. Summary Description of Randomized Clinical Trials Included in the Systematic Review (N = 41)

Author, year	Participants	n	Intervention	Dosage	Outcomes	Social well-being instrument	Follow-up (months)
**Alias, 2021**	Community-dwelling older adults	I = 194<br>C = 164	**I:** Physical and social group activities<br>**C**: Offered intervention at the end of the study	3m (1 per 1w/ 2h)	Nurse, GP, ER, inpatient visits (health records)	Social support (Social Resources Inventory	3, 12
**Asbury, 2011**	Women with cardiac syndrome x	I = 24<br>C = 25	**I:** Support groups<br>**C:** Standard care	12m (1 per 1m/ 90 min)	Self-reported GP visits	ENRICHD Social Support	6, 12
**Boter, 2004**	Stroke survivors	I = 263<br>C = 273	**I:** Individual support program<br>**C:** Standard care	6m (3 phone calls, 1 home visit)	Self-reported GP visits and readmissions	Social Support List–Discrepancies [SSL-D	6
**Buchkremer, 1995**	Caregivers of individuals with schizophrenia	I = 60<br>C = 31	**I:** Therapeutic self-help groups<br>**C:** Standard care + offered intervention at the end of the study	12m (1 per 2w)	Patients' re-hospitalisations (health records)	Number of social contacts	12
**Cheng, 2016**	Caregivers of stroke survivors	I = 64<br>C = 64	**I:** Individual psychoeducational program<br>**C:** Routine care - information leaflets on stroke and pre-discharge advice	3m (2 face-to-face meetings, 6 bi-weekly phone calls)	Stroke-related hospitalisations	Social support questionnaire (SSQ-6)	3
**Chien, 2010**	Caregivers of individuals with dementia	I = 46<br>C = 46	**I**: Multicomponent psychoeducational, case-management program<br>**C:** Routine family support services	6m (1 per 2w (total = 10)/ 2h)	Duration of residential placement	SSQ6	1w, 6, 12, 18
**Chien, 2013**	Caregivers of individuals with schizophrenia	I1 = 45<br>I2 = 45<br>C = 45	**I1:** Family-led mutual support group<br>**I2:** Psycho-education group<br>**C:** Standard psychiatric care	9m (14 group sessions / 2h)	Patients' re-hospitalisations (health records)	SSQ-6	1w, 12, 24
**Chien, 2014**	Individuals with mental illnesses	I1 = 36<br>I2 = 36<br>C = 35	**I1:** Mindfulness-based psychoeducation group<br>**I2:** Conventional psychoeducation group<br>**C:** Standard psychiatric care	3m (1 per 2w/ 2h)	Frequency and duration of readmissions to psychiatric hospital (health records)	SSQ-6	1w, 12, 24
**Chyzzy, 2019**	Pregnant adolescent women	I = 21<br>C = 19	**I:** Mobile phone-based individual peer support intervention<br>**C:** Standard community support services	3m (minimum of 10 contacts)	Self-reported visits to nurses, midwives, GP, ER & specialists	SSQ-6	3
**Colella, 2018**	Post-coronary surgery men	I = 61<br>C = 124	**I:** Telephone calls with peer supporters<br>**C:** Usual care: discharge teaching and booklet, routine check-ups	6w (1 per 1w)	Self-reported visits to GP, nurse, cardiologist, surgeon & ER	Funch's Shortened Social Support Scale (SSSS)	1.5, 3
**Constantino, 2005**	Family violence shelter residences	I = 13<br>C = 11	**I**: Support groups<br>**C:** Free-flowing group chat sessions	2m (1 per 1w/ 90 min)	The Health Screening Questionnaire	The Interpersonal Self Evaluation List (ISEL)	2
**Cui, 2019**	Individuals with heart failure	I = 48<br>C = 48	**I**: Educational self-management intervention<br>**C:** One group education session and information pamphlet	12m (1 per 4w/ 15-30 min)	Readmissions (health records)	The self-management ability - social support domain	12

© 2023 HaGani N et al. *JAMA Network Open.*

Author, year	Participants	n	Intervention	Dosage	Outcomes	Social well-being instrument	Follow-up (months)
**Dush, 2001**	Individuals with mental illnesses	I = 90<br>C = 92	**I**: Cognitive-behavioural group and home care<br>**C:** Routine care: psychiatric follow-ups and referrals to community mental health services	2w of prevention program and ongoing support	Number and duration of hospitalisations	The Shaw-Denmark Social Support Inventory	2w
**Franse, 2018**	Community-dwelling older adults	I = 986<br>C = 858	**I**: The Urban Health Centers Europe (UHCE) approach- multicomponent intervention<br>**C:** Usual care: access to existing care services	12m	Self-reported doctor visits & hospitalisation length	De Jong Gierveld loneliness scale	12
**Garvey, 2015**	Individuals with multimorbidity	I = 26<br>C = 24	**I**: Multicomponent group intervention<br>**C:** Offered intervention at the end of the study	6we (1 per 1w /3h)	GP, nurse and hospital VISIS (health records)	The Health Education Impact Questionnaire (HeiQ): Social integration and support scale	2
**Gaugler, 2015**	Caregivers of individuals with dementia	I = 54<br>C = 53	**I**: Individual and family counselling, support group<br>**C:** Biannual newsletter and quarterly "check-in" calls	4m (two individual sessions, 3 family sessions, support groups and counselling)	Residential care placements	The Stokes Social Network scale	4, 8, 12, 18, 24 [1]
**Gillard, 2022**	Individuals with mental disorder	I = 287<br>C = 291	**I**: Individual peer support<br>**C:** Standard care	4m (1 per 1w/ 1-1.5h)	Readmissions (health records)	The Social Contacts Assessment	4, 12
**Gleibs, 2011**	Older adults residing in care	I1 = 13<br>I2 = 11<br>C1 = 12<br>C2 = 9	**I1**: Group water clubs<br>**I2**: Individual activity focused on the benefits of water<br>**C1:** Group discussions daily life<br>**C2:** One-on-one sessions on daily life	2m (1 per 1w / 30 min)	Number of GP calls (health records)	Perceived social support	2
**Goldberg, 2013**	Individuals with mental disorder	I = 32<br>C = 31	**I:** Self-management peer-support<br>**C:** Standard care	3m (1 per 1w / 60-75 min)	Use of emergency department services	18-item Self-Management-access to social support scale	3, 5
**Grant, 2000**	Individuals with psychosocial problems	I = 90<br>C = 71	**I:** Referral facilitation to voluntary organisations<br>**C:** Standard care	1 assessment, 2-3 follow-ups	Number and cost of contacts with primary healthcare	The Duke-UNC functional social support questionnaire	1, 4
**Hengartner, 2017**	Individuals with mental disorder	I = 76<br>C = 75	**I:** Individual transitional intervention<br>**C:** usual care: assistance from a social worker during inpatient	3m (tailored to patients)	Frequency of readmissions, duration of inpatient stays according to medical records	F-SozU K-14 perceived social support	3, 12
**Hudson, 2012**	Pregnant adolescent women	I = 21<br>C = 21	**I**: New Mothers Network program: online psychological and informational resources<br>**C:** Usual care: parenting instructions	6m (resources available at all times)	Infants' clinic visits, and ER visits (self-reported)	Revenson's and Schiaffino's Social Support	1w, 6w, 3, 6

[1] Quarterly follow-up assessments during the first 12 months of participation and every 6 months thereafter for a minimum of 2 years or until the study ended, loss to follow up or death.

© 2023 HaGani N et al. *JAMA Network Open.*

Author, year	Participants	n	Intervention	Dosage	Outcomes	Social well-being instrument	Follow-up (months)
**Jiang, 2021**	Patients with chronic heart failure	I = 49<br>I2 = 57<br>C = 56	**I**: Self-management programme<br>**C:** Standard care<br>**I2:** Self-management programme + smartphone app	6w (1 per 2w)	Unplanned hospital admissions and medical consultations (health records)	SSQ-6	6w, 3, 6
**Jones, 2013**	Individuals with HIV	I = 83<br>I2 = 77	**I**: Monthly individual visits and group sessions<br>**I2:** One-on-one sessions	3m (1 per 1m / 90min)	Self-reported increased/decreased clinic visits	SSQ-8	3, 6
**Kidd, 2021**	Individuals with schizophrenia	I = 22<br>I2 = 41<br>C = 44	**I**: Peer support-delivered transitional intervention<br>**C:** usual care: referrals to outpatient case management and services<br>**I2:** I1 + 4 weekly visits (2h) after discharge	1w (1–2 meetings before discharge / 30–60 min and 1 visit (3h) after discharge)	Rehospitalisations and lengths of stay through the hospital electronic database	19-item Social Support Survey	1, 6
**Lindsay, 2009**	Individuals with coronary heart disease	I = 54<br>C = 54	**I:** New home computer and 1-year broadband subscription. Online group support, weekly drop-in sessions and phone support<br>**C:** New home computer and 1-year broadband subscription. Weekly drop-in sessions and phone support	9m	Visits to a GP, nurse, specialist and other healthcare providers in the past month	Social support questionnaire	6, 9
**Markle-Reid, 2003**	Older home care clients	I = 62<br>C = 64	**I:** Proactive nursing health promotion<br>**C:** Usual care: Home care services	6m (minimum of 1 per 1m/ 60 min)	Self-reported use of all types of health services	Personal Resource Questionnaire (PRQ-2000)	6
**Markle-Reid, 2021**	Community-dwelling older adults with multimorbidity and depressive symptoms	I = 47<br>C = 52	I: hospital-to-home transitional care intervention<br>C: Standard care	6m (1 per month)	Self-reported costs of use of primary care, emergency department and specialists, hospital days, other health	PRQ-2000	
**Morrell, 2000**	Post-natal women	I = 311<br>C = 312	**I:** Practical and emotional support<br>**C:** Routine visits by the community midwife	1m (up to 10 sessions/ 3h)	GP contacts, hospital services, NHS costs	Duke social support questionnaire	6w, 3, 6
**Muralidharan, 2019**	Individuals with mental disorder	I = 124<br>C = 118	**I:** Self-Management co-led peer support<br>**C:** Medical Illness Education and non-peer support group	3m (1 per week/ 75 minutes)	ER use (health records)	18-item Self-Management-access to social support scale	3, 6
**Reid, 2002**	Primiparous women	I = 503<br>C = 501	**I:** Support groups<br>**C:** Self-help manual (PACK)	3m (1 per 1w/ 2h)	Self-reported contact with health professionals	SSQ-6	3, 6
**Roberts, 1999**	Caregivers of individuals with cognitive impairment	I = 29<br>C = 29	**I:** Problem-Solving Counselling<br>**C**: Standard care	6m (up to 10 sessions)	Costs of primary care, emergency room and specialists, hospital days and other health professionals	The Duke Social Support questionnaire	6, 12

© 2023 HaGani N et al. *JAMA Network Open.*

Author, year	Participants	n	Intervention	Dosage	Outcomes	Social well-being instrument	Follow-up (months)
**Roncella, 2013**	Individuals with acute myocardial infarction	I = 49<br>C = 45	**I:** Short-term psychotherapy<br>**C**: Cardiac rehabilitation	Individual therapy: 3m (3-11 sessions/1h)<br>Group therapy: 3m (5 sessions/ 2h)	Rehospitalisations (health records)	Social Support Questionnaire	6, 12
**Shaw, 2016**	Caregivers of gastrointestinal cancer patients	I = 64<br>C = 64	**I:** Family Connect telephone intervention: emotional and practical support for caregivers<br>**C:** Usual care	2.5m (4 sessions)	Reported ER & readmissions	Caregiver Reaction Assessment	3, 6
**Simpson, 2014**	Individuals with mental disorder	I = 23<br>C = 23	**I:** Individual peer-support<br>**C:** Care as usual from community mental health services	4w	Client Service Receipt Inventory	The UCLA loneliness scale	1, 3
**Tarazona-Santabalbina, 2016**	Community-Dwelling Frail older adults	I = 51<br>C = 49	**I:** Supervised-facility multicomponent exercise program<br>**C:** Regular primary care program	6m (5 per 1week/ 65min)	ER and primary care visits	The Duke Social Support questionnaire	6
**Valencia, 2020**	Individuals with schizophrenia	I = 90<br>C = 86	**I:** Brief psychoeducation group for patients and fanilies<br>**C:** Standard care	1m (1 per week/ 2h)	Hospitalisations	WHOQOL-BREF- social domain	12
**Van Lieshout, 2018**	Community-dwelling pre-frail older adults	I = 139<br>C = 142	**I:** SPRY program interdisciplinary multicomponent intervention<br>**C:** Health and welfare services as usual; Invitation for the program after the study ended	6 months (11 meetings/ 1-2.5h)	Self-reported hospital/ nursing home admissions & primary care visits	De Jong Gierveld loneliness scale	3, 12
**VanLieshout, 2022**	Individuals with post-partum depression (PPD)	I =<br>C =	**I:** Group cognitive behavioural therapy<br>**C:** Standard care including PPD services such as psychotherapy	9w (1 per week/ 2h)	Self-reported mental healthcare visits	The Social Provisions Scale	2, 6
**Wiggins, 2004**	Post-natal at-risk women	I = 183<br>I2 = 184<br>C = 184	**I:** Supportive listening visits<br>**I2**: Community Group Support<br>**C:** 1 NHS health visiting and clinic support	12m (1 visit per 1m)	Self-reported GP, nurse, ER visits & hospitalisations	The Duke Social Support questionnaire	12, 18
**Yang, 2005**	Individuals with recurrent depression	I = 60<br>C = 61	**I:** Hospital-based psychosocial intervention-group and individual activities<br>**C:** Antidepressants	3m (daily)	Readmissions	WHO QOL-100- social relationships component	6, 12, 24

Abbreviations: **I**: Intervention group, **C**: Control group, **GP**: General practitioner, **ER**: emergency room, **min**: minutes, **h**: hours, **w**: weeks, **m**: months

© 2023 HaGani N et al. *JAMA Network Open.*

eTable 10. Grading of Recommendations Assessment, Development and Evaluation (GRADE) Evidence Profile

Outcome	№ of studies	Study design	Risk of bias [1]	Inconsistency [2]	Indirectness [3]	Imprecision [4]	Other consideration [5]	Overall certainty
HCU- OR	41	RCT	not serious	serious [a]	not serious	not serious	none	⨁⨁⨁◯ Moderate
HCU- OR (long-term effect)	18	RCT	not serious	serious [b]	not serious	serious [c]	none	⨁⨁◯◯ Low
HCU- SMD	38	RCT	not serious	serious [a]	not serious	serious [c]	publication bias strongly suspected [d]	⨁◯◯◯ Very low
HCU- SMD (long-term effect)	13	RCT	not serious	serious [b]	not serious	very serious [c]	publication bias strongly suspected [d]	⨁◯◯◯ Very low
Social support-SMD	33	RCT	not serious	serious [a]	not serious	not serious	none	⨁⨁⨁◯ Moderate
Loneliness-SMD	3	RCT	serious [e]	not serious [a]	not serious	serious [c]	none	⨁⨁◯◯ Low

Abbreviations: HCU: health care utilisation; **OR:** odds ratio; **SMD:** standardised mean difference; **RCT:** randomised control trial

Explanations

[a] $I^2 > 50\%$ $p < 0.05$

[b] $I^2 > 50\%$ $p > 0.05$

[c] Confidence intervals include outcomes with different clinical meanings

[d] Egger regression test $p < 0.05$

[e] Equal or greater than 50% of studies' weights are 'high risk'

[1] Risk-of-bias: certainty was downgraded if more than 50% of the weights of individual trials in each outcome assessed came from high-risk studies.

[2] Inconsistency: based on the variability and heterogeneity across individual trials. Certainty was downgraded if $I^2 > 50\%$ and/or if the p-value of the heterogeneity test was < 0.05.

[3] Indirectness: assessed qualitatively by the extent to which the population, interventions, and outcome measures directly reflect the aims of the systematic review.

© 2023 HaGani N et al. *JAMA Network Open.*

[4] Imprecision: based on inspection of the pooled estimate and the 95% confidence interval (95%CIs). Certainty was downgraded when 95%CIs included values that would have different clinical implications.

[5] Publication bias: assessed by a funnel plot and an extension to Egger's regression test. Certainty downgraded when $p < 0.05$.

© 2023 HaGani N et al. *JAMA Network Open.*

eTable 11. Strength of Evidence (Bayes Factors) in the OR and SMD According to Health Care Service

		Random effect (95%CI)	Bayes	Evidence of effect for H_1*
**OR**	**Primary care**	0.72 (0.52, 0.1.01)	3.62	Moderate
	**Inpatient**	0.74 (0.54, 1.02)	3.22	Moderate
	**Outpatient**	1.03 (0.72, 1.46)	0.40	Inconclusive
	**ER**	0.64 (0.43, 0.96)	5.56	Moderate
	**Total health care**	0.75 (0.59, 0.97)	5.63	Moderate
**SMD**	**Primary care**	-0.02 (-0.22, 0.18)	0.38	Inconclusive
	**Inpatient length**	-0.35 (-0.61, -0.09)	12.03	Strong
	**Inpatient**	-0.23 (-0.47, 0.00)	3.35	Moderate
	**Outpatient**	0.34 (0.05, 0.62)	6.99	Moderate
	**Total health care**	-0.10 (-0.29, 0.09)	0.84	Inconclusive
	**Social support**	0.25 (0.04, 0.45)	7.21	Moderate

***** Bayes factors determine how well the data are predicted by H_1 (the assumption that there is intervention effect) relative to H_0 (no intervention effect). 10< BFs < 30 indicates strong evidence for an effect; 10 > BFs > 3 indicates moderate relative evidence for an effect; 3 < BFs < 0.3 inconclusive evidence; and BF < 0.3 moderate evidence for no effect.

Abbreviations: OR: odds ratio; **SMD**: standardised mean difference; **CI**: confidence interval

eTable 12. Subgroup Analyses of Postintervention Health Care Utilization According to Participant- and Intervention-Level Factors

		SMD (95%CI)	OR (95%CI)
**Participant characteristics**			
**Age**	<30	0.02 (-0.32, 0.35)	0.93 (0.66, 1.32)
	30-60	-0.34 (-0.72, 0.04)	**0.61 (0.43, 0.86)**
	>60	0.00 (-0.30, 0.31)	0.99 (0.70, 1.39)
**Population group**	Caregivers	-0.45 (-0.95, 0.05)	**0.23 (0.07, 0.71)**
	Chronic illness	-0.01 (-0.42, 0.40)	0.94 (0.66, 1.34)
	Mental health	-0.42 (-0.91, 0.07)	**0.31 (0.13, 0.74)**
	Older adults	-0.10 (-0.48, 0.28)	1.07 (0.39, 2.94)
	Post-natal women	0.15 (-0.16, 0.47)	1.03 (0.88, 1.20)
	Violence survivors	-0.32 (-1.12, 0.49)	-
**Gender**	Men majority	-0.26 (-0.63, 0.10)	0.84 (0.44, 1.63)
	Women majority	-0.02 (-0.24, 0.20)	0.98 (0.44, 2.17)
**Country**	Australia	-	**0.23 (0.07, 0.71)**
	Canada	**0.58 (0.19, 0.97)**	0.94 (0.66, 1.34)
	China	**-0.44 (-0.68, -0.20)**	**0.31 (0.13, 0.74)**
	Columbia	-	1.07(0.39, 2.94)
	Europe (including the UK)	-0.07 (-0.22, 0.08)	1.03 (0.88, 1.20)
	USA	-0.22 (-0.65, 0.21)	**0.47 (0.32, 0.69)**
**Intervention characteristics**			
**Intervention type**	Group	-0.13 (-0.33, 0.07)	0.77 (0.56, 1.06)
	Individual	0.00 (-0.28, 0.28)	**0.74 (0.55, 0.98)**
**Components**	Single	-0.08 (-0.31, 0.16)	0.73 (0.53, 1.01)
	Multi	-0.14 (-0.48, 0.20)	0.77 (0.51, 1.16)
**Intervention delivery**	Coordinators	-0.04 (-0.44, 0.37)	0.74 (0.34, 1.64)
	Health professionals	-0.19 (-0.70, 0.32)	**0.65 (0.46, 0.93)**
	Peers	0.23 (-0.26, 0.72)	0.91 (0.56, 1.47)
**Intervention length**	Short (1-4 months)	0.15 (-0.09, 0.39)	**0.69 (0.51, 0.94)**
	Long	**-0.26 (-0.47, -0.06)**	0.87 (0.60, 1.26)

	SMD (95%CI)	OR (95%CI)
(6-12 months)		

Abbreviations: **SMD**: standardised mean difference, **OR**: odds ratio, **CI**: confidence interval

© 2023 HaGani N et al. *JAMA Network Open.*

eFigure 1. Cochrane Risk-of-Bias Summary of Low, High and Unclear Risk of Bias Across All Included Studies (N = 41)

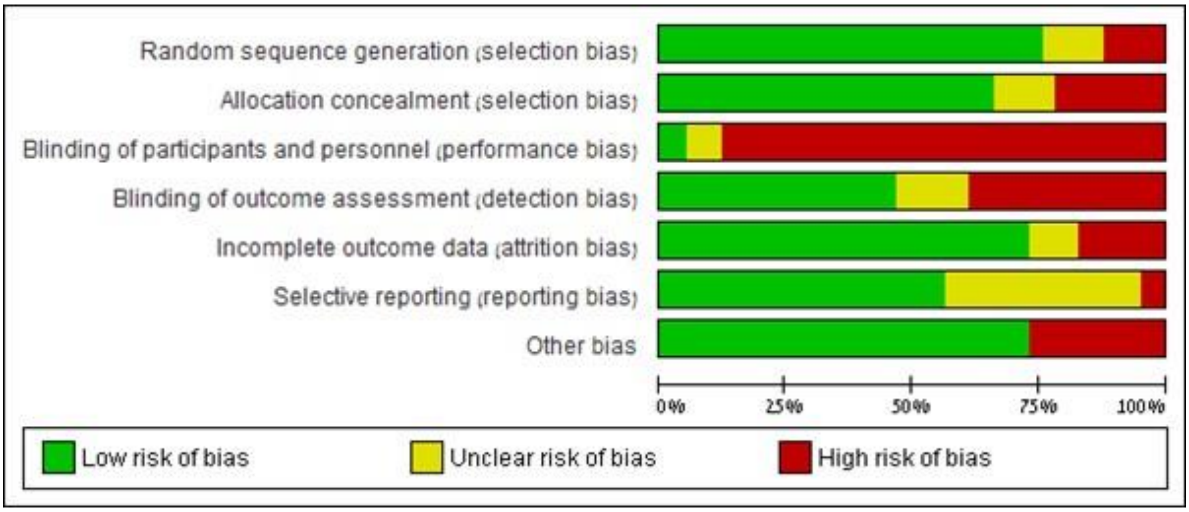

© 2023 HaGani N et al. *JAMA Network Open.*

eFigure 1b. Cochrane risk-of-bias summary: review authors' judgments about each risk of bias item for each included study (n = 41)

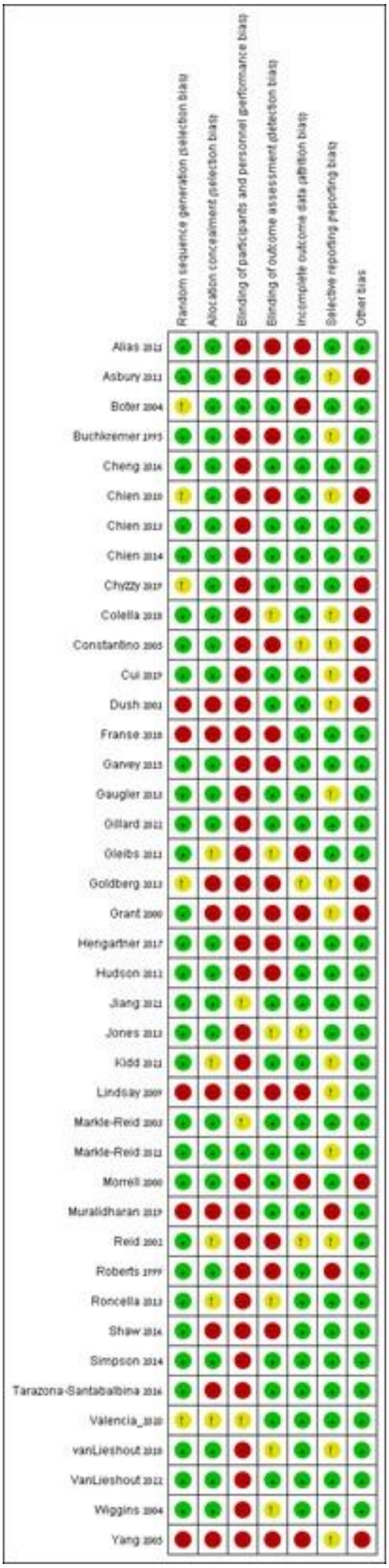

© 2023 HaGani N et al. *JAMA Network Open.*

eFigure 2. Publication Bias Funnel Plots for Standardized Mean Difference (SMD) and Log Odds Ratio (OR) Random Effect Models of Health Care Utilization Outcomes and Social Support

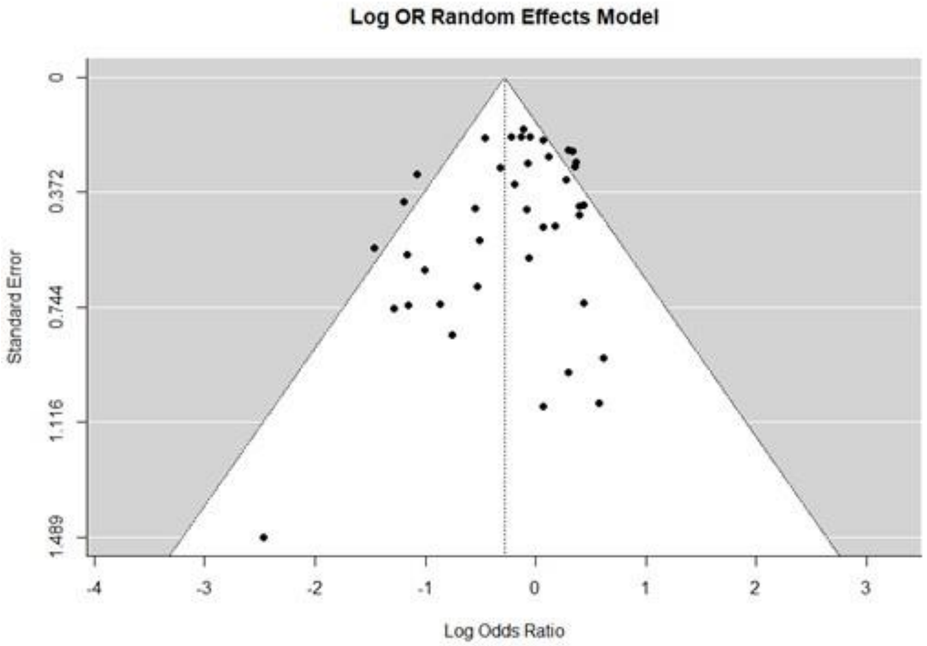

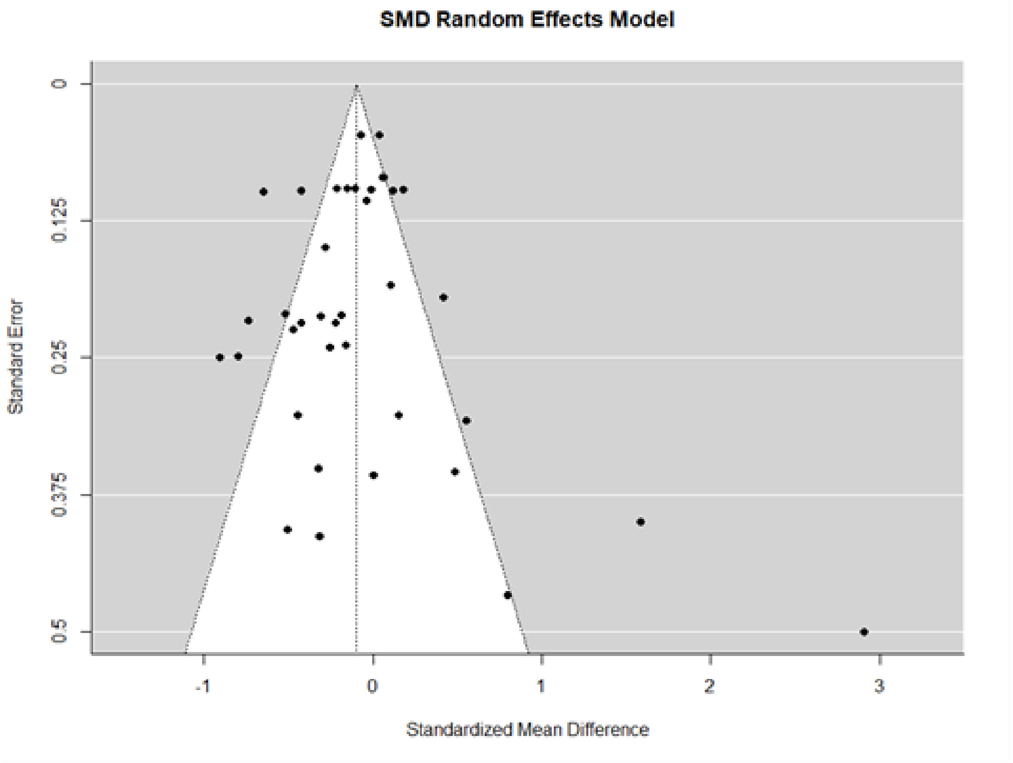

© 2023 HaGani N et al. *JAMA Network Open.*

eFigure 2b. Publication bias funnel plots for standerdised mean difference (SMD) and log odds ratio (OR) random effect models of sustained health care utilisation outcomes

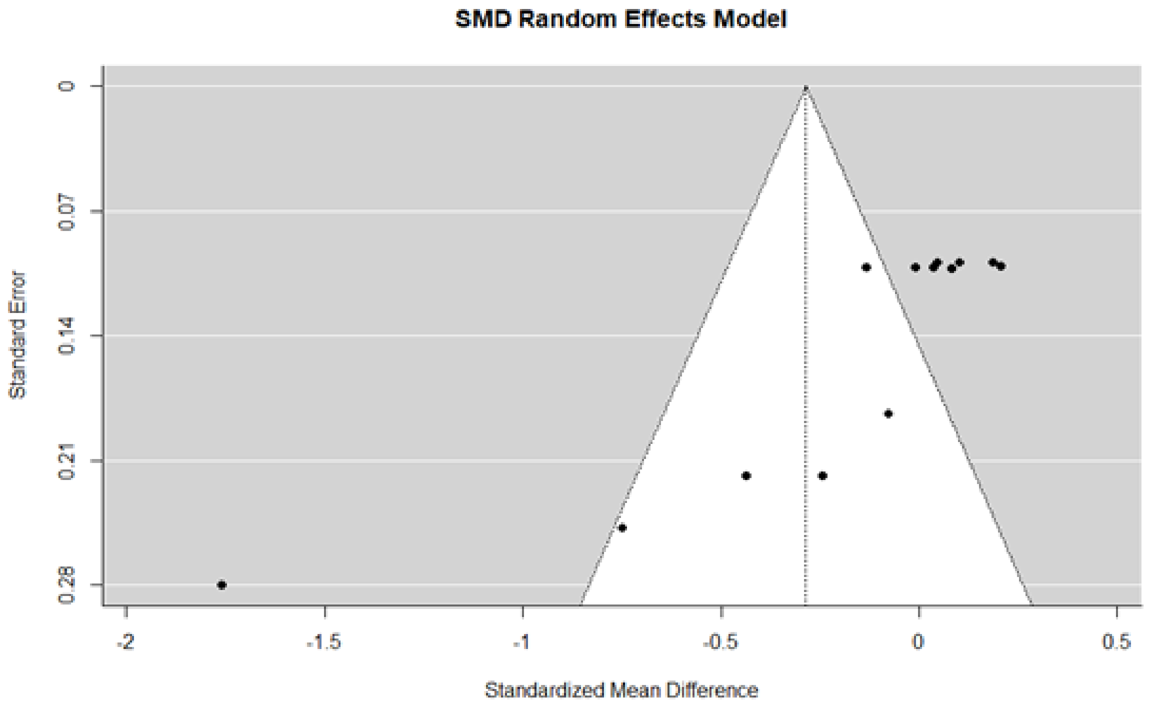

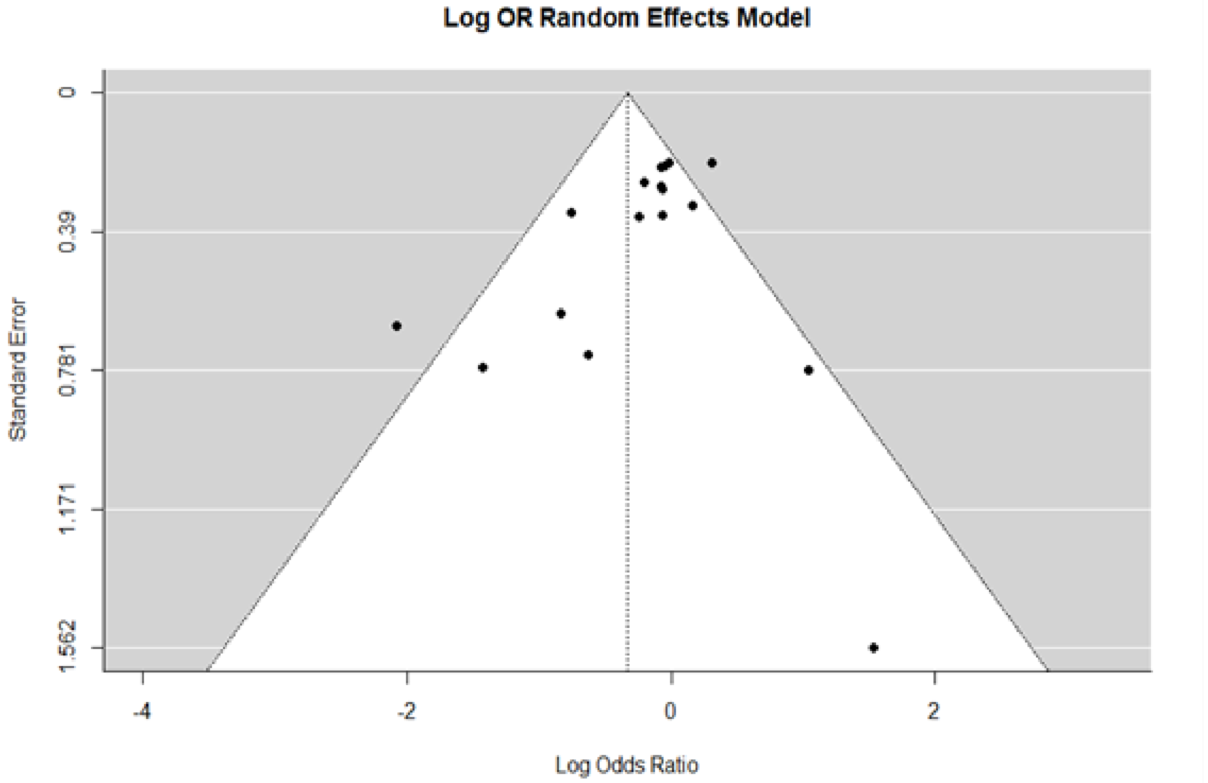

© 2023 HaGani N et al. *JAMA Network Open.*

eFigure 2c. Publication bias funnel plot for standerdised mean difference (SMD) random effect model of social support

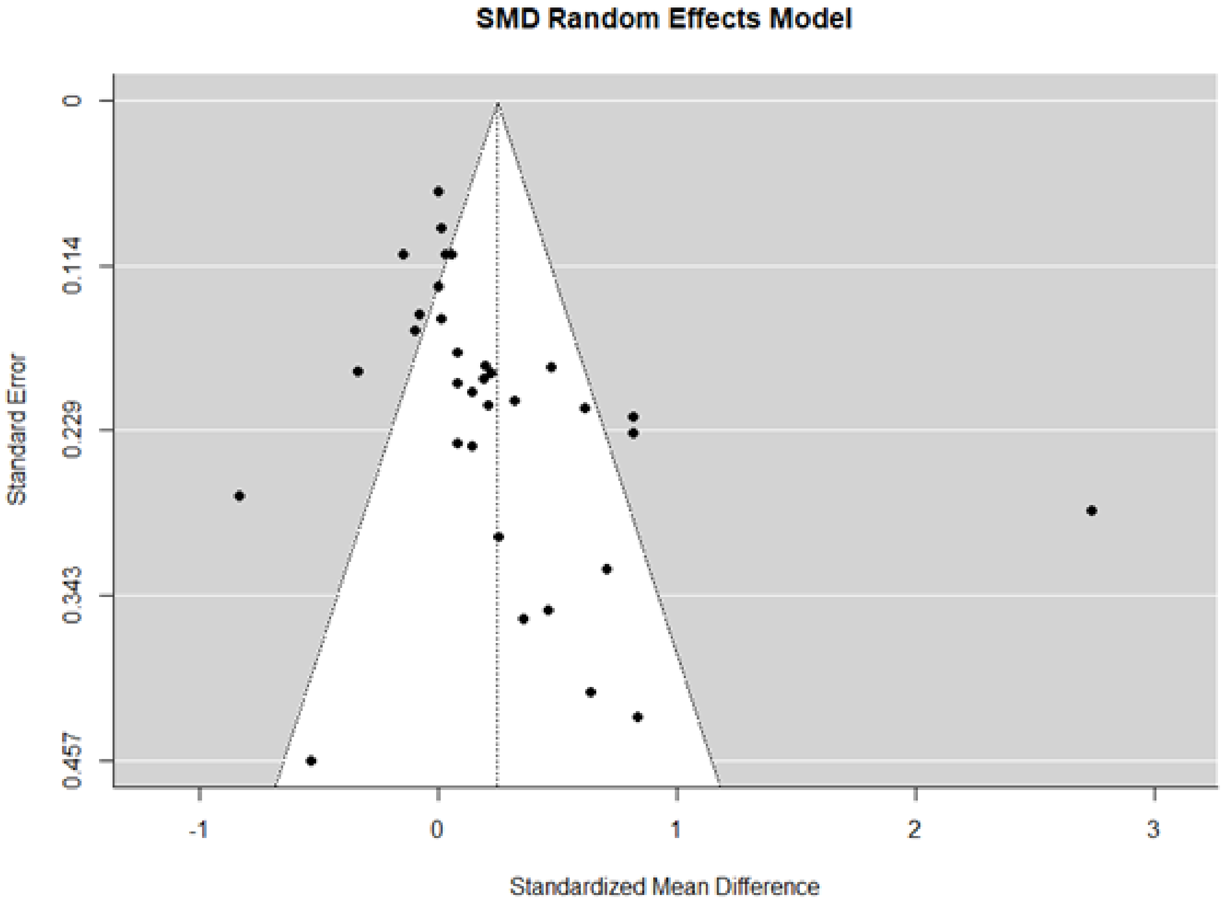

© 2023 HaGani N et al. *JAMA Network Open.*

eFigure 3. Pooled 95% CI Odds Ratio of the Sustained Association of Psychosocial Interventions With Health Care Utilization

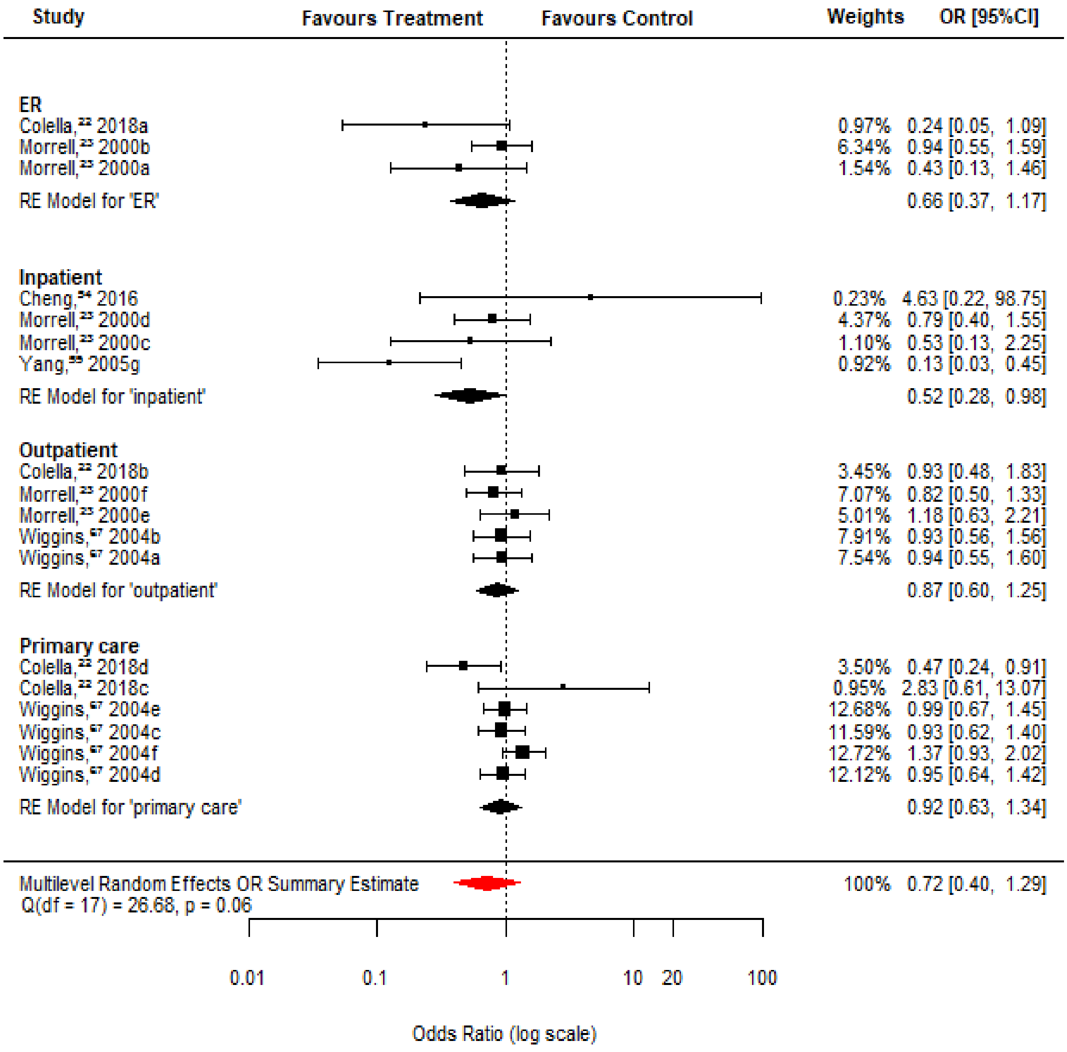

Abbreviations: ER: emergency room

© 2023 HaGani N et al. *JAMA Network Open.*

eFigure 4. Pooled 95% CI Standardized Mean Difference of the Sustained Association of Psychosocial Interventions With Health Care Utilization

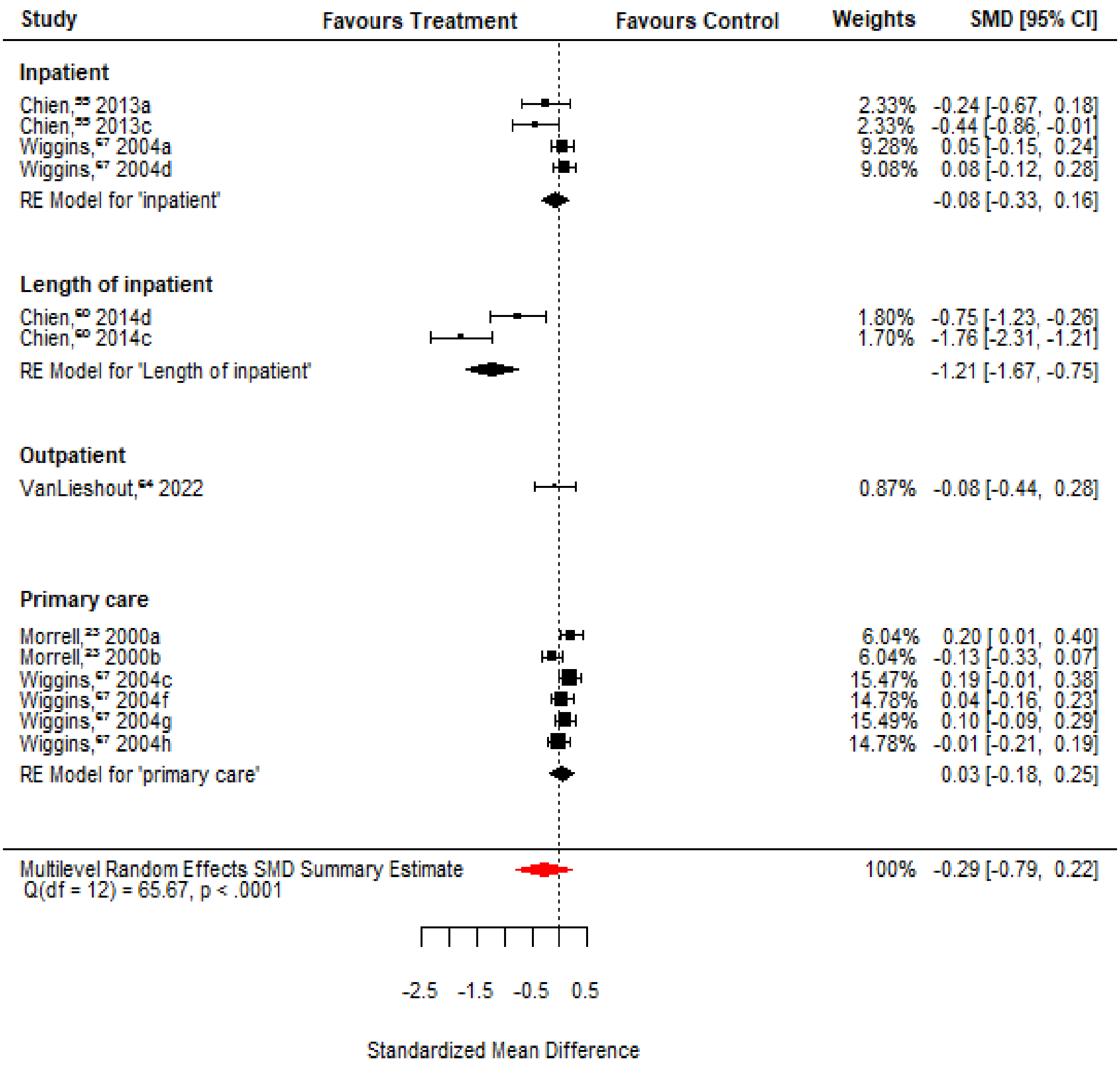

© 2023 HaGani N et al. *JAMA Network Open.*
