# Supplementary material for: Health Care Utilization Following Interventions to Improve Social Well-Being: A Systematic Review and Meta-analysis
Source: JAMA Netw Open. 2023 Jun 29;6(6):e2321019. doi: 10.1001/jamanetworkopen.2023.21019 (PMC10311391; doi:10.1001/jamanetworkopen.2023.21019)
Supplement: Supplement 2. — Data Sharing Statement [file jamanetwopen-e2321019-s002.pdf]

Data Sharing Statement

HaGani. Health Care Utilization Following Interventions to Improve Social Well-being. *JAMA Netw Open*. Published June 29, 2023. doi:10.1001/jamanetworkopen.2023.21019

Data
Data available: No

Additional Information
Explanation for why data not available: Data will be available upon request
